# Dynamic regulation of CTCF stability and sub-nuclear localization in response to stress

**DOI:** 10.1371/journal.pgen.1009277

**Published:** 2021-01-07

**Authors:** Bettina J. Lehman, Fernando J. Lopez-Diaz, Thom P. Santisakultarm, Linjing Fang, Maxim N. Shokhirev, Kenneth E. Diffenderfer, Uri Manor, Beverly M. Emerson

**Affiliations:** 1 Regulatory Biology Laboratory, Salk Institute for Biological Studies, La Jolla, California, United States of America; 2 Waitt Advanced Biophotonics Center, Salk Institute for Biological Studies, La Jolla, California, United States of America; 3 Razavi Newman Integrative Genomics and Bioinformatics Core, Salk Institute for Biological Studies, La Jolla, California, United States of America; 4 Stem Cell Core, Salk Institute for Biological Studies, La Jolla, California, United States of America; University of Cologne, GERMANY

## Abstract

The nuclear protein CCCTC-binding factor (CTCF) has diverse roles in chromatin architecture and gene regulation. Functionally, CTCF associates with thousands of genomic sites and interacts with proteins, such as cohesin, or non-coding RNAs to facilitate specific transcriptional programming. In this study, we examined CTCF during the cellular stress response in human primary cells using immune-blotting, quantitative real time-PCR, chromatin immunoprecipitation-sequence (ChIP-seq) analysis, mass spectrometry, RNA immunoprecipitation-sequence analysis (RIP-seq), and Airyscan confocal microscopy. Unexpectedly, we found that CTCF is exquisitely sensitive to diverse forms of stress in normal patient-derived human mammary epithelial cells (HMECs). In HMECs, a subset of CTCF protein forms complexes that localize to Serine/arginine-rich splicing factor (SC-35)-containing nuclear speckles. Upon stress, this species of CTCF protein is rapidly downregulated by changes in protein stability, resulting in loss of CTCF from SC-35 nuclear speckles and changes in CTCF-RNA interactions. Our ChIP-seq analysis indicated that CTCF binding to genomic DNA is largely unchanged. Restoration of the stress-sensitive pool of CTCF protein abundance and re-localization to nuclear speckles can be achieved by inhibition of proteasome-mediated degradation. Surprisingly, we observed the same characteristics of the stress response during neuronal differentiation of human pluripotent stem cells (hPSCs). CTCF forms stress-sensitive complexes that localize to SC-35 nuclear speckles during a specific stage of neuronal commitment/development but not in differentiated neurons. We speculate that these particular CTCF complexes serve a role in RNA processing that may be intimately linked with specific genes in the vicinity of nuclear speckles, potentially to maintain cells in a certain differentiation state, that is dynamically regulated by environmental signals. The stress-regulated activity of CTCF is uncoupled in persistently stressed, epigenetically re-programmed “variant” HMECs and certain cancer cell lines. These results reveal new insights into CTCF function in cell differentiation and the stress-response with implications for oxidative damage-induced cancer initiation and neuro-degenerative diseases.

## Author summary

Our tissues are subject to chronic physiological and environmental damage, yet little is known about how healthy human cells normally respond to stress. We examined the effect of damage on cells obtained from breast tissue of disease-free women. Unexpectedly, we identified a well-known protein regulator of chromosomal function, CTCF, as a robust target of stress signals. In normal mammary cells, a pool of CTCF is localized to large “depots” within the nucleus that regulate RNA processing. Upon cellular damage, CTCF rapidly disappears from nuclear “depots” by stress-inducible protein degradation while genome occupancy by CTCF is relatively unaffected. We observe the same phenomenon in neuronal progenitors differentiated from human pluripotent stem cells. We propose that in specific cell types, stress-sensitive forms of CTCF exist that have a unique function in RNA metabolism potentially by fine-tuning gene expression near nuclear speckles, which may maintain cells in a progenitor or adaptive state. Upon stress, this particular CTCF function is rapidly disabled, which may change the identity of cells most vulnerable to disease in order to safeguard them from becoming dysfunctional. Persistently stressed cells have lost this CTCF function, which may facilitate the genesis of damage-induced cancer initiation and neuro-degeneration.

## Introduction

Exposure of an organism or tissue to physiological stress results in an orchestrated cellular response that induces profound changes in gene expression, RNA processing, and protein synthesis that ultimately drive cell fate. This coordinated strategy accelerates adaptive processes necessary for individual cells within a population to survive diverse and unanticipated forms of stress [1]. Much of our knowledge about the human cellular stress response comes from studies in which human cancer cell lines were exposed to genotoxic agents. While valuable, these conditions do not adequately reflect how our normal healthy cells respond to physiological stressors that they are routinely subject to in vivo (e.g., hypoxia, inflammation, oxidative damage). Mechanisms that control the response and adaptation of normal human cells to stress and the process by which stressed cells return to homeostasis or become dysfunctional are largely unknown. To gain insight into these mechanisms, we investigated the stress response in primary human mammary epithelial cells (HMECs), which are derived from tissue obtained from disease-free women following voluntary surgical mammoplasty [2]. The HMEC cell culture system has been widely used to analyze how prolonged stress recapitulates early steps in aberrant epigenetic programming and genomic instability that are characteristic of human tumorigenesis [3–7]. Our aim was to use primary HMECs to investigate mechanisms underlying responses to both acute and chronic forms of cellular stress. Beyond the scope of our initial aim, our data unexpectedly revealed that the multi-functional nuclear protein CCCTC-binding factor (CTCF) is an exquisitely sensitive target of diverse forms of cellular stress.

The well-characterized protein CTCF has been shown to have diverse regulatory roles in transcription, epigenetic programming, and organizing 3D chromosomal architecture [8–14]. CTCF was first identified as a transcription factor [15,16] and subsequently shown to control gene imprinting and genetic domains through insulator/boundary DNA elements. Depending on the cell type and study, CTCF was reported to bind ~40,000–70,000 genomic DNA sites and act with proteins like Cohesin and non-coding RNAs to coordinate long-range inter- and intra-chromosomal interactions that may promote cell-type-specific transcriptional programs [11–14]. Primarily known to interact with DNA and chromatin, CTCF also associates with at least 17,000 endogenous RNAs through an RNA binding domain within the C-terminus that is distinct from its N-terminus 11-zinc finger DNA binding motifs [17–19]. CTCF-RNA interactions can either target or remove CTCF from specific regions of the X-chromosome to regulate X-inactivation or pairing [20,21]. CTCF also binds to a p53 antisense transcript to modulate p53 gene expression [22]. CTCF has been implicated in RNA processing by virtue of dynamically generating DNA loops that bring together different combinations of exons to generate RNA and protein diversity [23] and by slowing RNA polymerase progression to alter selection of exons in spliced RNAs [24–26]. CTCF is impacted by the cellular stress response where it can undergo deSUMOylation [27] and function as an anti-apoptotic factor in damaged cells [28,29]. CTCF has also been shown to limit oxidative stress by activating the Frataxin gene in endothelial cells and to promote vascular development [30]. Interestingly, CTCF is involved in the DNA damage response by facilitating DNA double-strand break repair [31]. Given the myriad activities of CTCF, environmental toxins could have adverse effects on its specific functions in the cellular stress response [32].

Our studies focused on defining early events in the response of normal cells to stress. Here we show that in patient-derived, cancer-free HMECs, CTCF is impacted by diverse forms of damage in a manner more consistent and sensitive than common stress markers, such as p53, ALDH1, and p16. Unexpectedly, we found that in normal HMECs, a pool of cellular CTCF localizes at nuclear speckles [33] where it associates with the serine/arginine-rich splicing factor SC-35 [34] and a common set of RNAs. This observation was validated by mass spectrometry analysis showing that nuclear speckle-associated proteins involved in RNA binding and splicing are CTCF interaction partners. Upon stress, this species of cellular CTCF is rapidly downregulated through protein degradation resulting in loss of CTCF from nuclear speckles and changes in RNA interactions shared between SC-35 and CTCF, yet with minimal perturbation of CTCF-genome interactions. Proteasomal inhibition restores these cellular CTCF protein levels and its localization to SC-35-associated nuclear speckles. Interestingly, many protein-coding RNAs that interact with CTCF and SC-35 in a stress-sensitive manner are implicated in cell differentiation or neuronal function. To explore this further, we analyzed the impact of stress signals on CTCF during neuronal differentiation of a human pluripotent stem cell (hPSC) model. Consistent with our observations in HMECs, stress-responsive CTCF complexes co-localize with SC-35-containing nuclear speckles but, unexpectedly, only at a particular stage of neuronal differentiation and not in differentiated neurons. We speculate that in HMECs and neural progenitor cells (NPCs), certain forms of CTCF have an adaptive function, which is rapidly regulated by stress, that maintains specialized cell states by modulating RNA diversity in response to changing environmental cues.

## Results

### CTCF is an exquisitely sensitive sensor of stress signals in normal patient-derived human mammary epithelial cells (HMECs)

**Fig 1:**
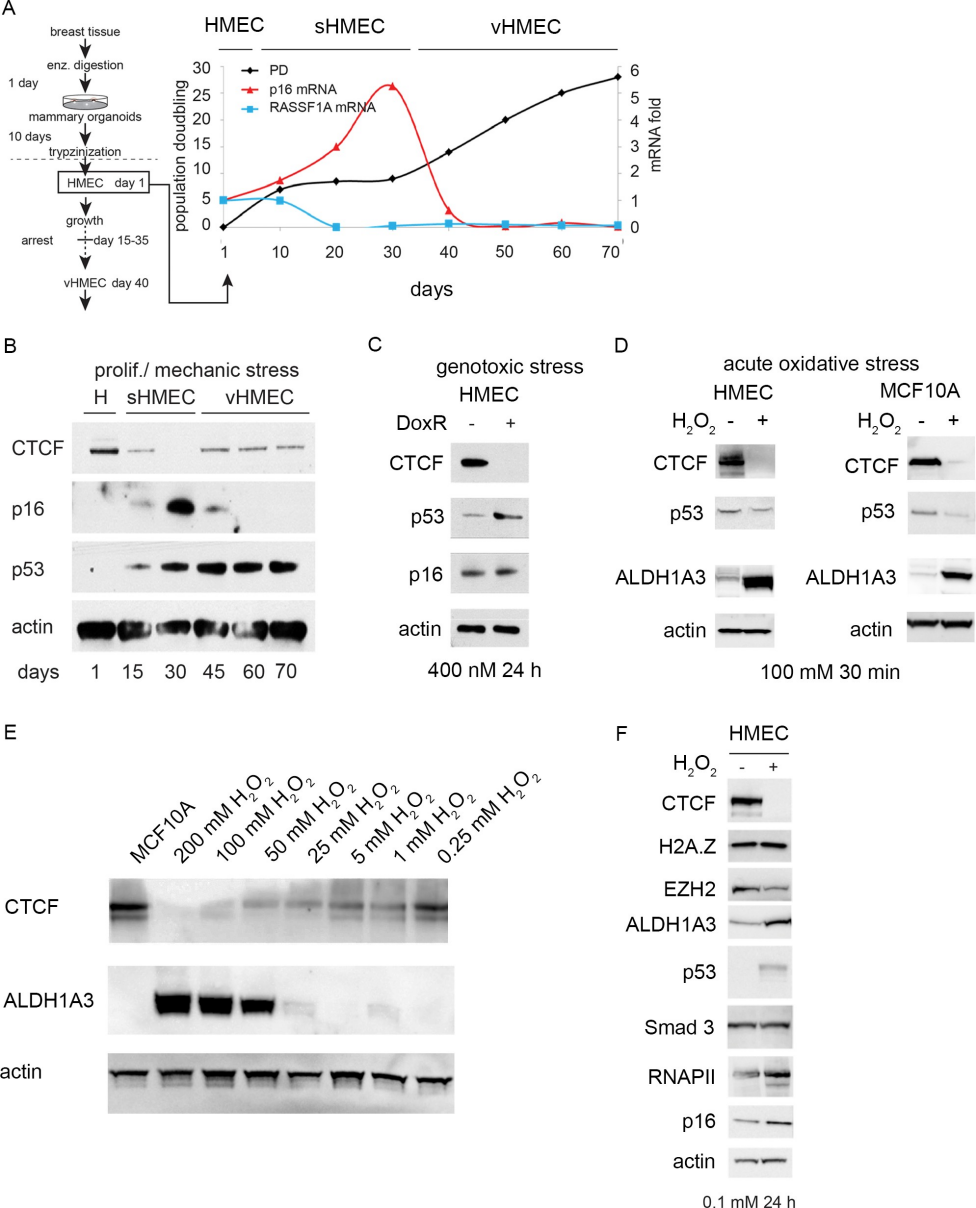
CTCF protein levels are selectively downregulated in normal epithelial cells upon stress. (A) HMEC isolation and cell culture diagram; population doublings and mRNA levels of tumor suppressors p16 and RASSF1A which are typically silenced in the classic HMEC/vHMEC primary culture model. Days in culture are indicated at the bottom. mRNA was measured by RT-qPCR and expressed as fold-change relative to day 1. (B) Protein extracts were prepared from patient-matched HMECs and vHMECs and analyzed by Western blot with the indicated antibodies. Representative Western blot analysis of CTCF, p16, p53, and actin levels in: HMECs (H); pre-selection, stressed HMECs (sHMECs); and post-selection vHMECs. (C) Western blot analysis of CTCF levels upon genotoxic stress by Dox (0.4μM, 24 hours) or (D) acute oxidative stress by H_2_O_2_ (100 mM, 30 min) in HMECs (day 1) and MCF10A cells. (E) MCF10A cells were treated with increasing concentrations of H_2_O_2_ over a period of 20 min to induce oxidative damage. Protein levels of CTCF, ALDH1A3, and actin (as a control) were assessed by Western blotting. (F) HMECs were treated with 0.1 mM H_2_O_2_ for 24 hours. Protein levels of CTCF, H2A.Z, EZH2, ALDH1A3, p53, Smad 3, RNA Pol II, p16, and actin were measured by Western blotting.

We analyzed the role of CTCF in both the acute and chronic stress response using primary cell cultures of normal HMECs. HMECs were propagated from disease-free reduction mammoplasty tissue from multiple healthy donors (age range 20–40 years old) chosen in an unbiased fashion. Epithelial-rich regions of tissue were dissected from surgical discard material and prepared as described [2,6,7], and as diagrammed (Fig 1A, left panel). With time in culture, a subpopulation of HMECs overcame the p16^INK4A^-induced senescence barrier and continued to proliferate (Fig 1A, right panel). Previous studies have shown that these “variant” HMECs (vHMECs) exist in a stably chronic stressed state, as indicated by elevated p53 protein levels, activation of the p53 target gene p21, and downregulation of p16 protein [6,35,36]. We extended this analysis by examining CTCF protein levels during a time course of the HMEC to vHMEC transition from days 1–70 (Fig 1B). We observed that CTCF protein levels were downregulated in normal HMECs by day 15 in culture in “stressed” but otherwise normal HMECs (sHMECs) before the p16-induced senescence barrier and remained stably repressed in proliferating vHMECs. We confirmed the stress-responsive nature of CTCF by showing that downregulation of CTCF protein levels in HMECs is induced by diverse forms of stress, such as the genotoxic agent doxorubicin (DoxR) (Fig 1C) and the acute oxidative stressor hydrogen peroxide (H_2_O_2_) (Fig 1D). Hydrogen peroxide also elevates the detoxification enzyme and cancer stem cell marker aldehyde dehydrogenase 1 family member A3 (ALDH1A3) [37,38] (Fig 1D). Importantly, it is critical to use freshly prepared HMECs because they rapidly become stressed and show elevated ALDH1A3 and decreased CTCF levels simply by freeze-thawing and prolonged tissue culturing. In fact, elevated expression of ALDH1A3 is a sensitive indicator of whether control cells are already stressed and have downregulated CTCF. Stress-induced downregulation of CTCF was also observed in spontaneously immortalized, non-tumorigenic human mammary epithelial MCF10A cells (Fig 1D and 1E), although not in other human breast cancer cell lines that we tested (S1 Fig). Care must be taken by using fresh, low-passage aliquots of MCF10A cells as they undergo a stress response accompanied by the appearance of multiple CTCF isoforms upon prolonged cell culture. With both HMECs and MCF10A cells, the normal, unstressed state should be carefully maintained in order to observe maximal CTCF stress-responsiveness. Notably, the impact of stress on other chromatin and transcription regulators is variable depending upon the stressor. For example, levels of the histone variant H2A.Z are unaffected by oxidative stress in HMECs (Fig 1F). In fact, we find that CTCF protein abundance in primary and non-tumorigenic mammary epithelial cells is consistently downregulated by all forms of stress that we have tested unlike other regulators and is far more sensitive to low levels of stressors than well-known stress markers such as p53 or ALDH1A3. Therefore, CTCF is a very robust sensor of the stress response in normal and non-tumorigenic human mammary epithelial cells, especially during acute stress. In fact, the abundance and nuclear distribution of CTCF in our experiments is a very sensitive indicator of whether primary cells are “normal” or not, as this state can be quickly lost under certain experimental conditions. An important consideration is that distinct MW forms of CTCF can be distinguished by immunoblotting using antibodies raised against different CTCF epitopes. For our Western blots, we used an N-terminal CTCF polyclonal antibody (Active Motif) that predominately recognized a protein(s) of ~110–120 kDa. The Active Motif CTCF antibody has been used to co-immunoprecipitate both hyper- and hypo-poly(ADP-ribosyl)ated (PARylated) forms of CTCF, which are each recognized by Western blot analysis with an N-terminal CTCF monoclonal antibody (G8; Santa Cruz) [39]. Given the biochemical and functional diversity of CTCF complexes, we expect that the stress-sensitive species reported here may represent a specifically modified form of CTCF.

### CTCF protein levels are downregulated by stress-induced changes in protein stability without significant alteration of CTCF-genome binding in HMECs

**Fig 2:**
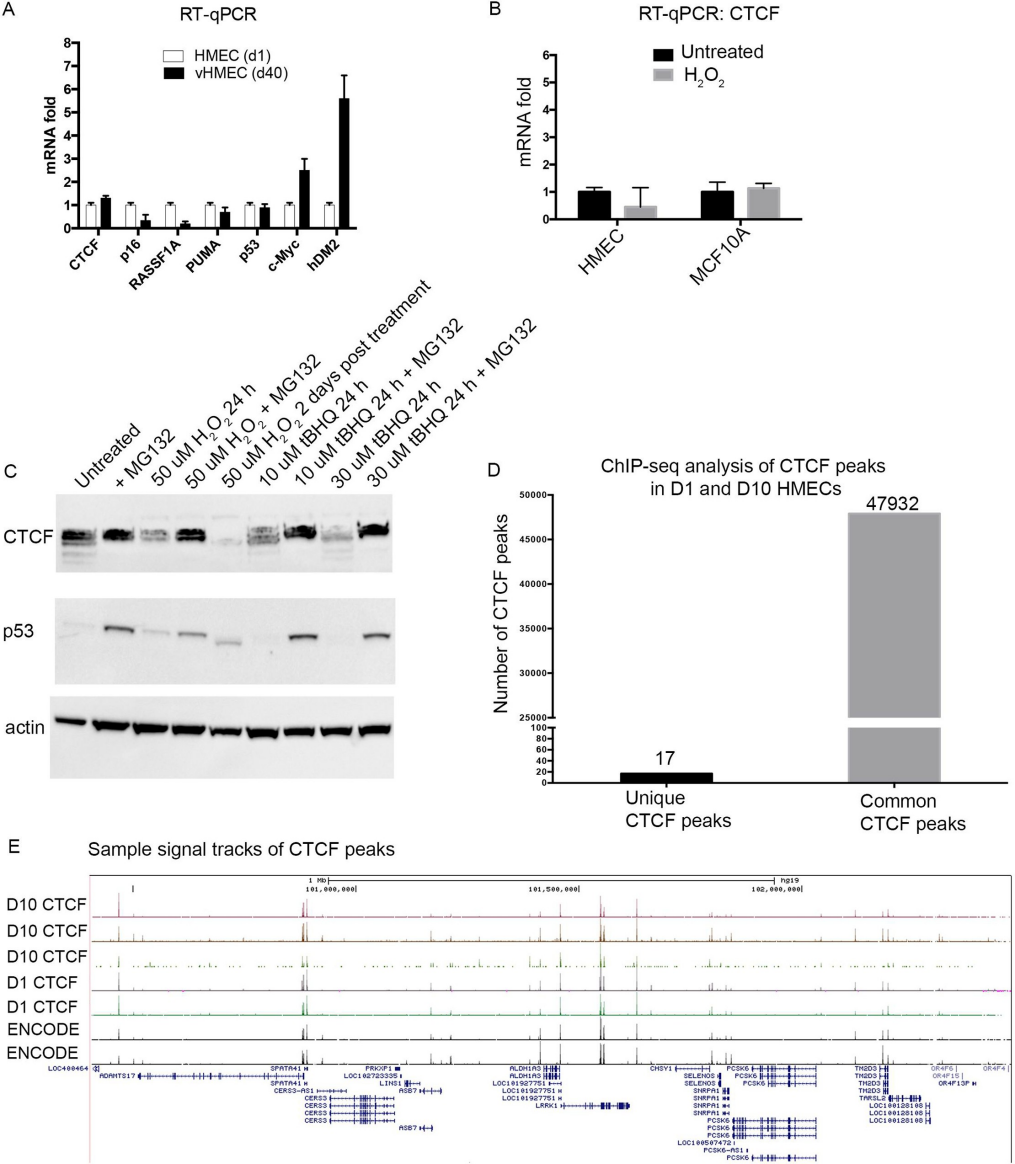
CTCF protein levels are downregulated by stress-induced changes in protein stability without significant alteration of CTCF-genome binding in HMECs. (A) Comparative mRNA levels of the indicated genes in vHMECs (day 40) relative to HMECs (day 1) as analyzed by RT-qPCR. (B) Comparative mRNA levels of CTCF upon H_2_O_2_ treatment in HMECs and MCF10A cells. (C) Oxidative stress in HMECs was induced with either H_2_O_2_ treatment for 24 hours or with the indicated concentrations of tBHQ. HMECs were stressed in the presence or absence of the proteasome inhibitor MG132. Protein levels of CTCF, p53, and actin were measured by Western blotting. (D) Chromatin immunoprecipitation ChIP-seq analysis of genome-wide CTCF-DNA binding in HMECs at day 1 and day 10. Shown are number of unique and common CTCF peaks. (E) ChIP-seq sample signal tracks of CTCF peaks demonstrate no changes in binding sites upon stress for the selected area.

To investigate possible mechanisms by which CTCF is so effectively downregulated, we measured CTCF mRNA levels in HMECs during prolonged cell culture stress (vHMECs) or by acute exposure to H_2_O_2_. Comparing levels of gene expression between HMECs and vHMECs revealed that CTCF and p53 mRNA levels were only slightly changed, whereas other genes were significantly downregulated (p16, RASSF1A) or elevated (c-MYC, HDM2) (Fig 2A). Similarly, oxidative damage induced in HMECs or MCF10A cells resulted in very little change in CTCF mRNA levels (Fig 2B). These results suggest that the decrease of CTCF protein in stressed HMECs and vHMECs is regulated at the levels of protein synthesis and stability. Indeed, treatment of HMECs with the inhibitor MG132 significantly elevated levels of CTCF, indicating a ubiquitin-proteasomal regulation of CTCF protein stability upon exposure to free radicals generated by H_2_O_2_ or tBHQ, similar to control of p53 protein stability (Fig 2C) [40]. We conclude that upon acute stress, a fraction of CTCF, detectable with the specific antibodies we used, is rapidly downregulated by changes in protein stability.

Since CTCF is a critical regulator of chromatin architecture, we investigated how such a decrease in protein levels of this stress-responsive form of CTCF may affect occupancy of the almost 48,000 CTCF genomic binding sites in HMECs during the stress response [41]. The reduction in cellular CTCF protein levels observed upon stress in normal HMECs suggested that the interaction and function of this regulator with a subset of its numerous genomic sites were likely to be impacted. For example, RNAi-mediated downregulation of CTCF in certain cancer cell lines results in destabilization of chromatin boundary elements and aberrant epigenetic silencing of specific tumor suppressor genes [42]. We therefore expected to see substantial changes in the interaction of CTCF with a subset of its numerous genomic sites in HMECs during the stress response. To this end, a comparative ChIP-seq analysis of genome-wide CTCF-chromatin interactions in HMECs at day 1 and day 10 was performed. Limited changes in global CTCF interactions were observed (0.04% changes at a threshold of adjusted p-value <0.05), despite a significant stress-induced decrease in abundance of CTCF cellular protein in the range of ~120 kDa. As shown in the barplot, the number of differential CTCF peaks in HMECs between day 1 and day 10 was only 17 among 48,000 total peaks (Fig 2D). Comparative signal tracks of CTCF peaks between multiple samples from HMECs at day 1 and day 10 align with each other and with ENCODE data (Fig 2E). Thus, CTCF may facilitate the stress response in normal epithelial cells by modulating its binding to low-affinity genomic sites or through other activities [43]. Interestingly, live cell single molecule imaging studies have shown that ~20–50% of CTCF is not DNA-bound but freely diffusing with rapidly exchanging genome binding that is dynamically regulated throughout the cell cycle [44–47]. Recent studies show that CTCF may be transiently sequestered in small nuclear “zones” through its RNA-binding region to more efficiently locate its genomic binding sites [48]. In normal HMECs, stress signals that rapidly and reversibly modulate CTCF protein stability and nuclear localization may also impact its dynamic, exchanging DNA-interactions to facilitate the cellular damage response.

### Proteomic analysis of CTCF interaction partners identifies nuclear speckle- associated proteins involved in RNA processing

**Fig 3:**
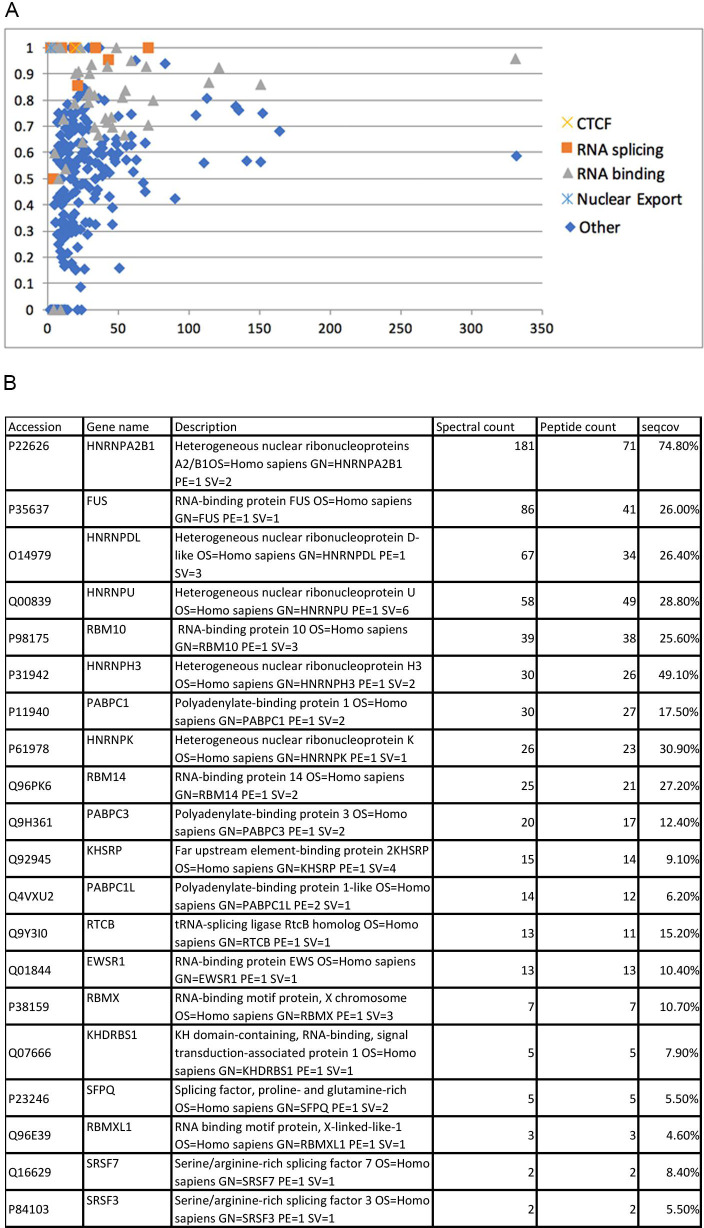
Analysis of functional annotation clustering for CTCF mass spectrometry hits. (A) DAVID Bioinformatics Resources 6.7 was used to analyze functional annotation clustering results from the mass spectrometry hits for CTCF cellular interaction partners, highlighting enrichment of RNA splicing and RNA binding proteins. (B) Table of top-scoring relevant CTCF mass spectrometry hits.

To investigate the possible function of stress-responsive CTCF complexes in HMECs, we conducted a mass spectrometry analysis of CTCF protein interaction partners. Since we were unable to isolate sufficient amounts of endogenous CTCF from patient samples, we used spontaneously immortalized, non-transformed mammary epithelial MCF10A cells as a source of CTCF. Similar to HMECs, MCF10A cells show stress-dependent downregulation of CTCF protein and CTCF nuclear localization (Figs 1D, 1E and S2). We planned to compare CTCF complexes in both normal and stressed MCF10A cells. However, due to the significant decrease in CTCF abundance, we were unable to obtain enough CTCF protein from stressed cells for mass spectrometry analysis. Thus, we focused on analyzing CTCF interaction partners in normal MCF10A cells to reveal the variety of interaction partners and potential function of CTCF complexes. Immunoprecipitated CTCF complexes were analyzed by mass spectrometry (Q Exactive mass spectrometer (Thermo) and Integrated Proteomics Pipeline–IP2 (Integrated Proteomics Applications). Identified proteins were filtered using DTASelect [49] with a target-decoy database search strategy to control the false discovery rate to 1%. The list of CTCF interaction partners yielded 314 IDs with a Homo sapiens background (S1 Table). To extract biological features associated with the identified mass spectrometry hits, DAVID Bioinformatics Resources 6.7 (DAVID; NIAID, NIH, http://david.abcc.ncifcrf.gov) was applied to analyze functional annotation clustering. This revealed that CTCF interaction partners are enriched in proteins involved in RNA biology, including RNA binding, RNA splicing, and nuclear export (Fig 3A) as indicated by the top-scoring hits (Fig 3B).

Many previous studies have identified numerous RNA binding partners for CTCF and demonstrated their critical role in regulating CTCF-dependent processes [14,17–22]. We also detected RNA binding and RNA splicing proteins (including snRNPs, hnRNPs, serine-arginine proteins) as the most prevalent CTCF interaction partners which predominantly localize to inter-chromatin SC-35 nuclear speckles [50,51]. CTCF has been proposed to regulate alternative RNA splicing through several different mechanisms [24,52–54], some of which may be co-translational in close association with chromatin. Thus, a CTCF-dependent activity at SC-35 nuclear speckles may serve to fine-tune processing of RNA transcribed from genes within its vicinity [50]. CTCF is known primarily to interact with chromatin-associated proteins and these new partners may reveal new functional roles for this diverse regulator.

### CTCF forms stress-responsive complexes at SC-35 nuclear speckles in normal HMECs

**Fig 4:**
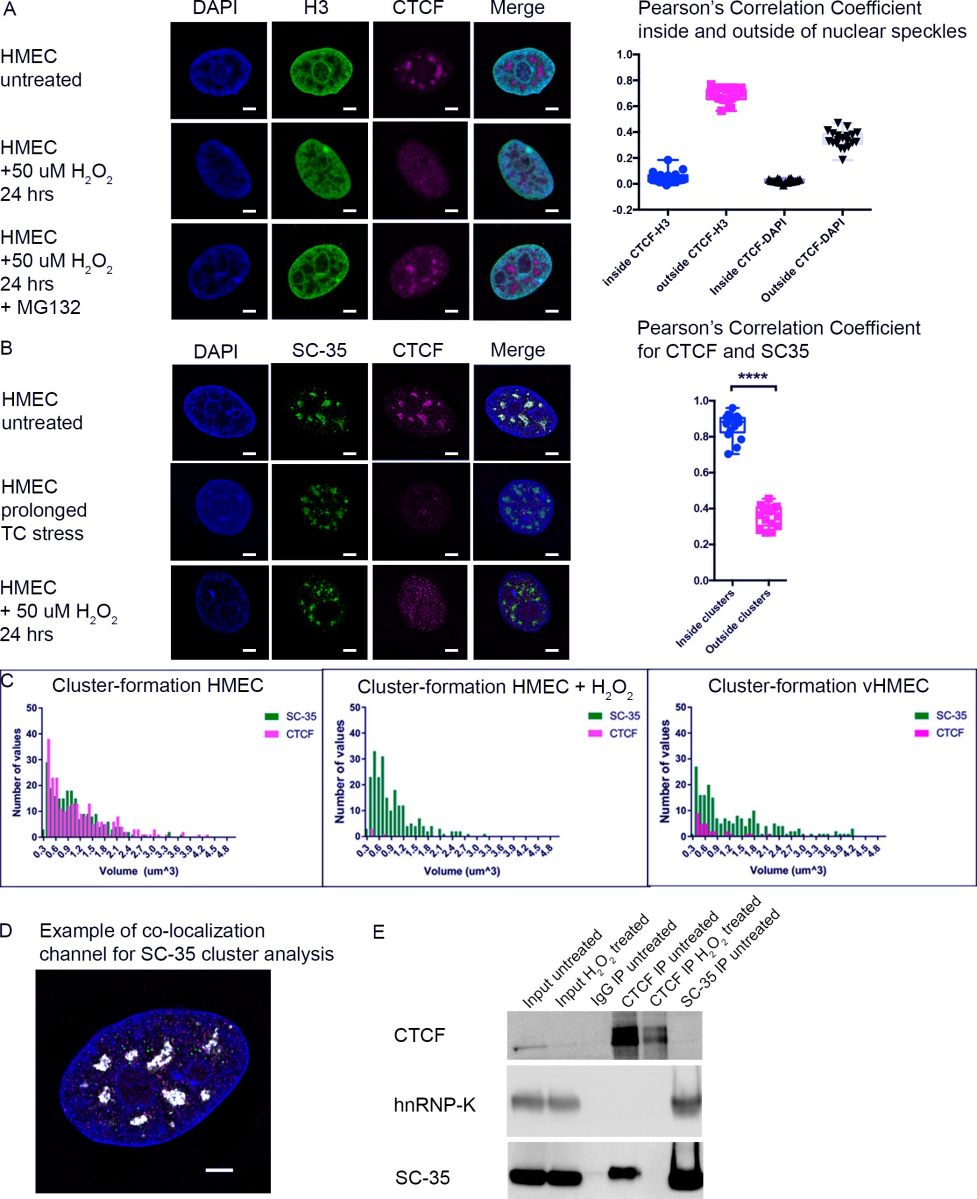
CTCF forms stress-sensitive complexes at SC-35 positive nuclear speckles in normal patient-derived HMECs. (A) HMECs were exposed to H_2_O_2_ treatment in the presence or absence of the proteasome inhibitor MG132 and stained against anti-CTCF and anti-histone H3 antibodies as well as DAPI to visualize the nuclear DNA. Scale bar = 3 μm, (HMEC untreated: n = 20; HMEC + 50 uM H_2_O_2_ 24 hours: n = 20; HMEC + 50 uM H_2_O_2_ 24 hours + MG132: n = 27). (B) HMECs were exposed to cell culture stress, or H_2_O_2_ treatment, fixed and stained against antibodies to CTCF and nuclear speckle marker SC-35 and DAPI, (HMEC untreated: n = 20; HMEC prolonged TC stress: n = 20; HMEC + 50 uM H_2_O_2_ 24 hours: n = 23). Samples were visualized with Airyscan microscope and the Pearson’s coefficients for co-localization were determined using Imaris software; 4B showed a significant difference (****, P<0.0001). (C) Histograms demonstrate the presence of CTCF-associated clusters (red) in unstressed HMECs and the loss of CTCF-cluster association in H_2_O_2_-treated HMECs and in stably stressed vHMECs. By contrast, the distribution of SC-35 in nuclear speckles (“clusters”) (green) remains relatively stable across the cell samples. (D) Example of co-localization of CTCF with SC-35 inside and outside of nuclear speckles. (E) Western blot shows SC-35 co-immunoprecipitation with CTCF (CTCF IP untreated), while no SC-35 was co-immunoprecipitated with CTCF under stressed conditions (CTCF IP H_2_O_2_-treated). The control co-immunoprecipitation of hnRNP-K (as a protein known to reside in nuclear speckles) with SC-35 and a small amount of CTCF confirmed the SC-35 speckle identity.

To explore the potential involvement of CTCF in RNA processing in nuclear speckles (Fig 3), we visualized its sub-nuclear distribution in HMECs using Airyscan confocal microscopy (Fig 4). Our observation of downregulation of CTCF protein levels upon cellular stress prompted us to simultaneously examine whether CTCF nuclear localization was also affected by stress signaling. Previous studies have shown that stress signals can promote growth arrest accompanied by changes in differential CTCF poly(ADP-ribosyl)ation (PARylation) and genomic binding. In normal tissues and breast cancer cells, PARylated CTCF predominates upon cell cycle arrest but transitions to a hypo-PARylated form in proliferating cells, which is also associated with breast cancer progression [55,56]. This important work shows that cellular stress and cell cycle stage transitions can evoke significant changes in CTCF post-translational modifications, which modulates site-specific genomic occupancy and gene expression. We performed imaging in three independent experiments (from three different patient tissue specimens) using unstressed HMECs, HMECs acutely exposed to H_2_O_2_, or HMECs stressed by prolonged cell culture (vHMECs). We first analyzed co-localization of CTCF with histone H3 and DAPI, as a DNA marker. Intriguingly, this revealed that CTCF was enriched in large nuclear bodies within the inter-chromatin space that is excluded from DAPI and H3 staining (confirmed by a Pearson’s Correlation Coefficient close to zero). By contrast, the minority of CTCF signal was detected outside the inter-nuclear space in a punctate pattern highly associated with histone H3, as expected for genome-associated CTCF (Pearson’s Correlation Coefficient ~0.7) (Fig 4A). However, we cannot rule out interactions between CTCF and H3 at the border due to the signal detection threshold. Consistent with our observation that oxidative stress significantly downregulates a certain pool of CTCF through changes in protein stability (Fig 2C), the large CTCF inter-chromatin bodies disappeared upon exposure to H_2_O_2_ stress, leaving only the punctate, H3-associated fraction of CTCF detectable (Fig 4A). However, the large CTCF-associated nuclear bodies could be partially restored by inhibiting proteasome-mediated degradation with MG132 and elevating CTCF cellular abundance in stressed HMECs (Fig 4A). Formation of large nuclear clusters by CTCF has been described previously in a pioneering study by Zirkel and colleagues. They showed that decreased expression of the high-mobility group protein, HMGB2, initiates cellular senescence by inducing pronounced spatial redistribution of CTCF into nuclear depots with concomitant genomic reorganization [57]. This and other studies underscore the importance of large-scale changes in CTCF nuclear localization in potentially driving fundamental cellular processes like senescence, the cell cycle, and the cellular stress response.

To further investigate possible functions of stress-responsive CTCF inter-nuclear bodies, we analyzed their co-localization with other known nuclear markers. Nuclear bodies can concentrate factors like proteins, DNA, RNA, and/or lipids in reaction sites for specific biological processes, or segregate these factors away from unwanted sites [33,50,51]. Our previous [58] and current proteomic analyses identified a variety of RNA processing proteins as CTCF interaction partners (Fig 3). These included SC-35, a marker for nuclear speckles, which are inter-chromatin domains enriched in pre-mRNA splicing factors [50,51]. To examine whether stress-responsive CTCF protein co-localized with SC-35 in HMECs, we imaged CTCF both inside and outside of the SC-35-associated nuclear speckles in normal and stressed HMECs. Airyscan microscopy confirmed that the species of CTCF detected by the antibodies we used predominantly co-localized to the SC-35-positive nuclear speckles in normal HMECs (highly significant Pearson’s Correlation Coefficient). Upon chronic (vHMECs) or acute (H_2_O_2_) stress, CTCF clusters disappeared from the SC-35 nuclear speckles. However, SC-35 association with nuclear speckles remained stable in stressed cells despite the loss of CTCF, indicating that CTCF is not required to maintain the structure of these nuclear bodies (Fig 4B and 4C). A co-localization channel is illustrated for SC-35-positive cluster analysis (Fig 4D). SC-35 is associated within the same complexes as CTCF in HMECs and its interaction is regulated by stress signals, as shown by co-immunoprecipitation experiments (Fig 4E).

Several nuclear bodies have RNA-associated functions, such as mRNA retention, RNA splicing, or RNA export [33,50,51]. To determine whether the CTCF-SC-35 associated nuclear speckles contain RNA compartments, we combined fluorescently-labeled oligo d(T) FISH probes with immune-CTCF staining. Indeed, co-localization of CTCF nuclear speckles with poly(A) RNA was observed. CTCF also co-localizes with SC-35 nuclear speckle-associated proteins [59], PABPN1, which binds to nascent poly(A) tails, and hnRNP-K, an RNA processing protein, but not to NONO, a paraspeckle-specific marker (S2 Fig). Since CTCF has been shown to associate with nucleolar components, like Nucleolin [42], we examined and eventually excluded nucleoli as a co-localization site for the stress-responsive CTCF-associated speckles (S2 Fig). Nuclear speckles are a major nuclear body, yet their function is not clearly understood. Recent genomic mapping data revealed that a significant chromosomal compartment is in close proximity with nuclear speckles, suggesting that RNA processing in nuclear speckles may be tightly coupled to transcribing genes [50].

Taken together, these results implicate a role for CTCF in stress-responsive RNA biology at nuclear speckles in primary HMECs. The fact that SC-35 nuclear localization was unchanged in H_2_O_2_-treated cells suggests that certain forms of CTCF are specifically targeted for degradation by the stress response. We did not observe CTCF interaction with nuclear speckles in patient-matched fibroblasts derived from the same breast tissue (S1 Fig). Moreover, in the breast cancer cell lines that we examined CTCF is no longer responsive to stress-induced protein downregulation nor enriched at SC-35 nuclear speckles (S1 Fig). To validate our results and exclude the possibility that post-translational modifications (PTM) might obscure CTCF-antibody recognition sites, we used antibodies against two different epitopes of CTCF outside of known PTM sites (N-terminus, Active Motif; C-terminus B-5, Santa Cruz) and observed similar patterns of sub-cellular localization (S3 Fig). This highlights the unique function and biological relevance of CTCF during the stress response in primary human epithelial cells and suggests that this specific role of CTCF is a target of deregulation.

### CTCF-RNA interactions in normal and stressed HMECs reveal functional roles in cell development and neuronal differentiation

**Fig 5:**
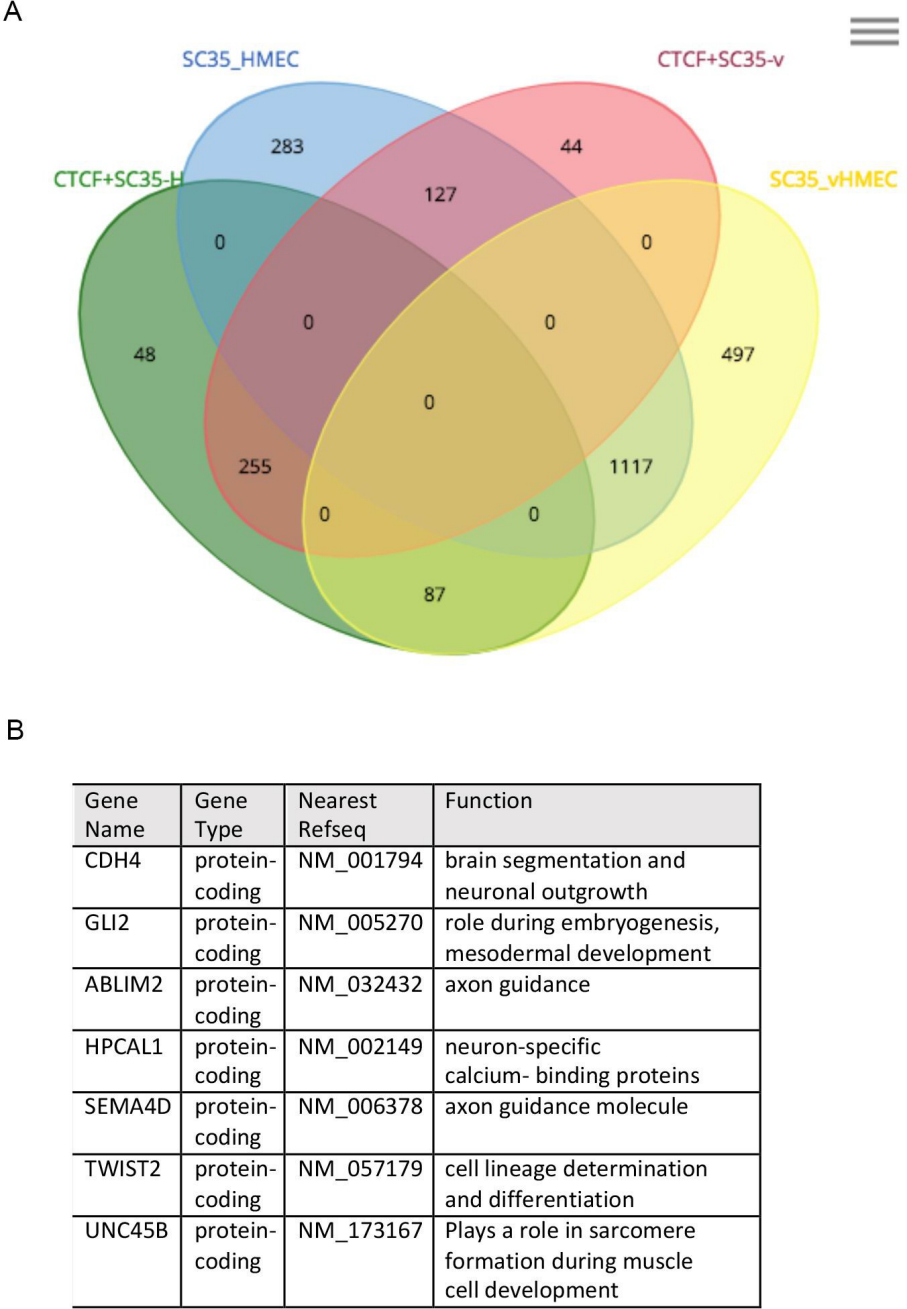
Stress-sensitive CTCF-RNA interactions that are shared with SC35. (A) Venn diagram showing the number of unique RNAs interacting with CTCF and/or SC-35 in HMECs compared to vHMECs, based on RIP-seq data. (B) List of top CTCF interactions with RNA in HMECs that were lost in vHMECs, but still remained bound to SC-35.

The observed nuclear co-localization of CTCF with poly(A) RNA and splicing factors at SC-35 nuclear speckles in normal HMECs prompted us to identify stress-responsive changes in the landscape of CTCF-bound RNA targets. To this end, we performed RNA-protein immunoprecipitation (RIP) analyses in HMECs with either CTCF-specific or SC-35-specific (as a positive control for RNA at the nuclear speckles) antibodies and sequenced the RNA recovered from the immunocomplexes (RIP-seq) [60,61]. Input samples were also collected and sequenced for each cell type to determine input over specific RIP. To select for enriched RNAs that specifically associate with CTCF or SC-35 in HMECs but dissociate in vHMECs (correlated with stress-induced downregulation of CTCF at SC-35 nuclear speckles), we compared RNAs in samples obtained by an identical procedure from vHMEC extracts. Our RIP-seq analysis revealed that 360 different RNAs were bound to both SC-35 and CTCF in HMECs (Fig 5A, S2 and S3 Tables). Despite the profound loss of CTCF protein abundance and depletion from the SC-35 nuclear speckles, we identified only 48 genes that no longer interacted with CTCF in vHMECs but still remained bound to SC-35. These data demonstrate that the majority of RNAs that associate with SC-35 and CTCF remain unchanged, despite stress-induced downregulation of a particular CTCF pool and its depletion from nuclear speckles in vHMECs. We cannot rule out that low levels of CTCF in vHMECs remain at the nuclear speckles, sufficient to detect RNA interactions by RIP-seq, or that other forms of CTCF not detectable by the antibodies we used interact with SC-35 outside of the nuclear speckles. Nevertheless, a role for CTCF in specific alternative splicing events, RNA transport or nuclear export in either HMECs or vHMECs remains a possibility.

We were also intrigued by the gene ontology (GO) term analysis for RNAs that are significantly bound by CTCF and SC-35 but dissociate upon stress. Surprisingly, this analysis revealed that most protein-coding RNAs that initially interacted with both CTCF and SC-35 in HMECs but lost their CTCF interaction in vHMECs, are involved in cell development/differentiation or neuronal function (Fig 5B). For example, genes involved in brain segmentation and neuronal outgrowth (CDH4) [62], axon guidance (ABLIM2, SEMA4D) [63,64], and cell lineage development (GLI2, TWIST2, UNC45B) [65–67].

Our nervous system is routinely exposed to different sources of oxidative stress, including free radicals from the redox potential of neurotransmitters, high levels of lipids as an oxidation substrate, and an elevated requirement for oxygen, which generates free radicals as by-products [68]. An inefficient response to oxidative stress in the developing brain or in differentiated neurons may compromise the nervous system leaving it vulnerable to neurodegenerative disorders [69,70]. Furthermore, a controlled oxidative stress response can influence the proper development of our nervous system, while the intracellular redox regulation and sensitivity to such stress varies between the embryonal stage, neural progenitor cells, and differentiated neurons [71,72]. This prompted us to investigate potential roles for CTCF in both the regulation of neural development and the response to oxidative stress at SC-35 nuclear speckles.

### Stress-responsive CTCF complexes at SC-35 nuclear speckles form during a specific stage of human neuronal differentiation

**Fig 6:**
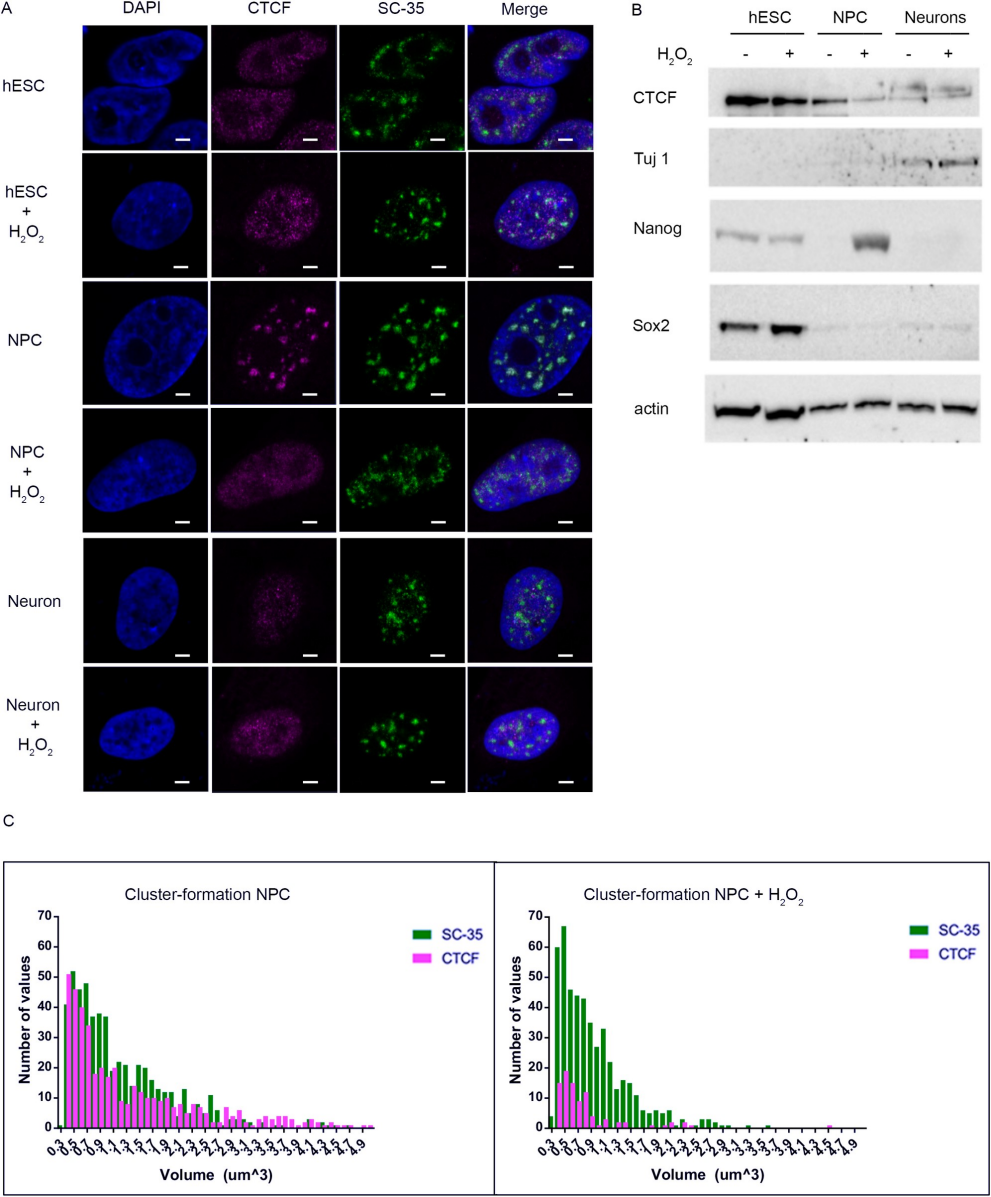
Stress-sensitive CTCF complexes at SC-35-associated nuclear speckles are exclusively found at the neural progenitor cells (NPCs) stage of neuro development. (A) hPSCs, NPCs, and early neurons were exposed to H_2_O_2_ treatment and stained with anti-CTCF, anti-SC-35 and DAPI. Samples were visualized with an Airyscan microscope; (NPC: n = 21; NPC + H_2_O_2_: n = 21). (B) hPSCs, NPCs, and early neurons were exposed to H_2_O_2_ treatment and CTCF protein levels were analyzed by Western blotting. Developmental markers for different neuronal stages are also shown. (C) Histograms demonstrate loss of CTCF cluster-formation (red) from NPCs to H_2_O_2_-treated NPCs, whereas the distribution of SC-35-associated clusters (green) remains relatively stable.

To gain insight into possible functions of CTCF during neural differentiation, we analyzed its stress-sensitivity and nuclear distribution pattern in human pluripotent stem cell (hPSC)-derived neural progenitor cells (NPCs) and early neurons. Cell identities were assessed using previously established criteria [73]. Using immunofluorescence, the nuclear distribution of endogenous CTCF during neuronal differentiation and targeting to nuclear speckles were determined by co-immunostaining with antibodies against CTCF and SC-35. Airyscan imaging revealed that CTCF was diffusely distributed in the nuclei of both undifferentiated hPSCs and neurons (Fig 6A). Strikingly, in NPC nuclei CTCF associated with large clusters and a strong Pearson’s correlation coefficient was observed for co-localization of CTCF and SC-35, similar to the pattern in unstressed HMECs. CTCF and SC-35 co-localization was significantly reduced in pluripotent and differentiated cells, suggesting that the functional interaction between CTCF and SC-35 nuclear speckles is specific to a particular stage of neural differentiation. We also examined the stress-sensitivity of CTCF nuclear localization at different stages of neural differentiation by visualizing normal and oxidative-stressed cells using Airyscan imaging. Consistent with our previous results in HMECs, stress signals induced profound changes in CTCF nuclear localization only when associated with nuclear speckles in NPCs whereas CTCF distribution in hPSCs and neurons was unaffected. As previously reported, CTCF protein levels detected by the specific antibodies we used markedly decrease as pluripotent hPSCs undergo differentiation into a variety of lineages [74]. We found that the abundance of this species of CTCF protein is high in hPSCs (like HMECs) but decreases in NPCs and is very low in differentiated neurons. Interestingly, stress-induced downregulation of this CTCF species occurs exclusively in NPCs, consistent with our imaging results, showing stress-sensitive CTCF co-localization at SC-35 nuclear speckles (Fig 6B). In pluripotent stem cells and in neurons, the protein abundance of this form of CTCF and its nuclear distribution are stable to stress.

Taken together, these data suggest that CTCF has the potential to serve a unique functional role which is highly responsive to cellular stress by associating with SC-35 nuclear speckles at a specific stage of neural development. In this capacity, CTCF complexes containing SC-35 and specific RNAs may maintain neural progenitor states through adaptable switches in RNA processing or fine-tuned gene regulation within speckles but rapidly lose this function during the stress response. Several recent studies have revealed important functions for CTCF in nerve regeneration and neuronal differentiation. One study showed that conditional loss of CTCF impaired the regenerative capability of sensory neurons by altering epigenetic programming and the transcriptional response to injury [75]. Intriguingly, CTCF protein abundance was shown to regulate a differentiation switch between myelinogenesis and Schwann cell maturation to promote myelin regeneration and Schwann cell myelination after nerve injury [76]. Underpinning the rapid, functional plasticity of CTCF observed during differentiation and environmental responses, is the finding that CTCF-genomic binding dynamics play a critical role in modulating gene expression frequency through stochastic gene choice to drive differentiation of neuronal progenitors to mature neurons [77].

## Discussion

CTCF is a well-known master regulator of gene expression, epigenetic programming and 3D genomic architecture. The diverse functions of CTCF have been attributed to its physical association with thousands of genomic DNA binding sites to organize and regulate chromatin through genetic control elements, such as insulators and boundaries, and topologically associating domains (TADs) [8–10,12,14]. Most of our information about CTCF biology is based on studies using cancer cell lines, embryonic stem cells or fibroblasts under unstressed conditions. Given the central importance of CTCF in a myriad of genomic functions, we investigated the role of CTCF in the stress response of normal human cells. We chose to use primary HMECs and oxidative stress as a widely applicable physiological stressor, which has been implicated in the aging process and diseases, such as cancer and neurological disorders [68,71,78].

Our studies revealed that a specific pool of CTCF protein is a very responsive sensor of diverse forms of stress in normal human epithelial cells. Cellular levels of this form of CTCF protein are quite high in normal HMECs, unlike patient-matched fibroblasts, but decrease dramatically upon acute exposure to oxidative or genotoxic stress and remain stably downregulated in persistently stressed, epigenetically re-programmed “variant” HMECs (vHMECs). The rapid loss of this CTCF species is due to a stress-induced change in protein stability, which is reversible by inhibition of proteasome-mediated degradation, suggesting a link between CTCF ubiquitination and stress signals. In the human cancer cell lines that we examined, CTCF protein levels were quite variable depending upon the cell line and levels were not affected by stress. This suggests that the ability of stress signals to modulate the abundance and function of a certain pool of CTCF is deregulated in the cancer cell lines that we tested.

Interestingly, Docquier and colleagues previously noted variable levels of CTCF protein abundance in breast cancer cell lines and normal breast tissues when detected by a C-terminal CTCF polyclonal antibody (Abcam). They hypothesized that increased levels of CTCF may protect breast cancer cells from apoptosis. Indeed, they found that experimentally decreasing levels of CTCF protein in breast cancer cell lines resulted in increased apoptosis whereas CTCF overexpression afforded partial protection against cell death. These authors proposed that CTCF overexpression may be a compensatory mechanism that evolved as a selective advantage to promote breast cancer cell survival by impeding apoptosis [79]. We have also observed quite variable CTCF protein abundance in primary HMECs, fibroblasts, cancer cell lines, and during differentiation of human pluripotent stem cells (hPSC), using primarily an N-terminal CTCF polyclonal antibody (Active Motif). However, it remains a possibility that the CTCF antibody used in our studies does not detect all species or modified forms of CTCF. Moreover, knockdown of CTCF in all cell types we examined resulted in significantly decreased cell survival. We have not observed a strict correlation between CTCF protein abundance and cell survival but agree that this is likely to be very dependent upon cell context.

Recent studies have defined critical roles for CTCF in the cellular stress response [32] and the importance of post-translational modifications that are known to affect CTCF activity, most notably poly(ADP-ribosylation) (PARylation) [80], phosphorylation [81], and SUMOylation [82] (diagrammed in S3 Fig). One study showed that upon genotoxic damage in cancer cell lines, CTCF is rapidly recruited to DNA damage sites via interaction of zinc finger DNA binding motifs with poly (ADP-ribose) (PAR) moieties [83]. This report also showed that CTCF-deficient human osteosarcoma U2OS cells rendered them hypersensitive to genotoxic stress. Seminal work by Hilmi and colleagues demonstrated that CTCF facilitates the DNA damage response specifically through the homologous recombination-mediated DNA double-strand break repair pathway. CTCF was shown to initially bind to DNA double-strand breaks independent of PARylation and subsequently recruit BRCA2 in a (PARylation)-dependent manner [31]. Other interesting studies by Lu and colleagues showed that stress-induced apoptosis of human corneal epithelial and hematopoietic myeloid cells was accompanied by downregulation of CTCF RNA and protein, mediated at the transcriptional level by the NF-kB pathway upon stimulation by epidermal growth factor (EGF) [28,29]. Manipulation of CTCF levels in these cell types indicated that decreased CTCF by knock-down promoted apoptosis whereas elevated CTCF by ectopic expression suppressed apoptosis. Further, this group demonstrated that hypoxic and oxidative stresses induce CTCF de-SUMOylation, possibly affecting CTCF regulation of its downstream target genes [27]. By contrast, our results unexpectedly reveal that rapid downregulation of a particular pool of CTCF by diverse stressors is controlled at the level of protein stability by proteasome-mediated degradation. Taken together, previous reports and our data highlight the significance of post-translational modifications in controlling the stability and stress-responsiveness of CTCF in a cell-type and context-dependent manner. Another important consideration is the likely adverse impact of environmental toxins on the diverse functions of CTCF and how this might contribute to impaired biological processes and degenerative diseases [32].

Despite the decrease in CTCF protein abundance in HMECs undergoing the stress response, our ChIP-seq analysis revealed only minor changes in global CTCF-genome binding (only 0.04% changes at a threshold of adjusted p-value <0.05) when compared to normal unstressed HMECs. Our results with a natural, stress-induced partial “knock-down” of a particular CTCF protein species are consistent with reports using artificial degron-mediated CTCF depletion in mouse embryonic stem cells which showed a general stability of CTCF-genome binding and chromatin organization even upon significant loss of CTCF protein [84]. Another study, also using degron-mediated degradation of CTCF in mouse ESCs, showed that CTCF looping and insulation of TADs is dependent upon CTCF protein abundance whereas active and inactive genome compartments remained stable even when CTCF was depleted [85]. This suggests that regulation of CTCF protein levels may be another way to modulate its genome binding dynamics. CTCF-genome interactions are partially conserved with an overlap of 40–60% CTCF binding across all tested cell types (HeLa, Jurkat, and CD4+ T cells) [86] and up to 70% similarity of genome-wide CTCF occupancy between cell types [41]. However, a subset of CTCF binding sites is capable of actively responding to the external environment and dictating cell-type specific differentiation and development [11,46,87]. CTCF dissociation from a stable chromatin interaction site could result in permanent epigenetic alteration of adjacent loci. Therefore, it may be advantageous for a cell to maintain a constant genomic architecture through stable CTCF binding, while a subset of CTCF recognition sites are dynamically regulated to respond to stress and other physiological signals. Indeed, a substantial amount of CTCF (~20–50%) has been shown by single-molecule imaging to be freely diffusing and to have rapidly exchanging interactions with cohesin at co-occupied chromatin sites that anchor TADs [44,46]. CTCF actively dissociates from chromatin and rapidly re-distributes to small clusters at other genomic sites, indicating that CTCF-mediated chromatin looping and TADs are in flux throughout the cell cycle [45,47]. Our results suggest that CTCF dynamics can also be regulated by rapid changes in protein stability induced by cellular stress signals, which likely impacts specific CTCF functions. It is also possible that some forms of CTCF with particular PTMs may be resistant to stress-induced degradation.

Since a particular form of CTCF protein is significantly degraded in HMECs upon stress yet only 0.04% of CTCF-genomic binding changes, we further analyzed CTCF cellular distribution during the stress response using high-resolution microscopy. Surprisingly, we observed that high levels of a specific pool of CTCF protein in normal HMECs is localized to SC-35-associated nuclear speckles. Upon stress, CTCF within speckles is degraded while CTCF-genome interactions are relatively unchanged, as measured by ChIP-seq. Although, it is possible that stress-regulated CTCF-genome interactions occur at the speckle periphery. Previous work has shown that ~20–50% of CTCF is freely diffusing and rapidly exchanging with genome-bound CTCF [44–47]. This suggests that stress-sensitive forms of CTCF may have different post-translational modifications or cofactors that render it a target of ubiquitin-mediated proteolysis or that degradation of freely diffusing CTCF occurs stochastically.

CTCF protein interaction partners have been identified using mass spectrometry or yeast two-hybrid approaches as well as by comparing genomic CTCF recognition sites that overlap with other DNA binding proteins. To date, the major classes of CTCF cofactors are annotated as proteins involved in binding and modification of DNA or chromatin, including DNA-binding proteins (YY-1, Cohesin), DNA and RNA helicases, BRG1, poly (ADP-ribose) polymerase (PARP-1), nucleolin, nucleophosmin, topoisomerase II, and transcription factor TFII-I [58,88,89]. Our mass spectrometry analysis revealed the most prevalent CTCF interaction partners to be those engaged in RNA binding and RNA splicing. These top hits predominantly localize to interchromatin SC-35 nuclear speckles. Our mass spectrometry results complement Airyscan images that localize stress-sensitive CTCF at SC-35 nuclear speckles in both normal HMECs and in hPSC-differentiated neural progenitors. The discrepancy with other published studies regarding CTCF nuclear localization is most likely due to the use of different cell types, such as immortalized cancer cell lines, or embryonic stem cells, where we also did not detect CTCF enrichment at SC-35 nuclear speckles.

Previous studies have shown that the sub-nuclear distribution of CTCF is dynamic throughout the cell cycle [45,47], suggesting that it has different functional roles. For example, CTCF re-localizes to the nucleolus during growth arrest in apoptotic and differentiated cells in several experimental systems. Interestingly, the 180 kDa poly(ADP-ribosyl)ated isoform of CTCF is targeted to the nucleolus through its central zinc finger domain where it appears to inhibit RNA polymerase I-dependent transcription [90]. Other work has revealed that in breast cancer cells, cell cycle genes are regulated by estrogen receptor-mediated interactions with CTCF localized to the nuclear lamina [91]. In a seminal study, Zirkel and colleagues revealed the fundamental role of the high mobility group B protein, HMGB2, and its linkage to CTCF in regulating chromatin re-organization in human primary cells undergoing senescence. Specifically, HMGB proteins are known to influence the boundaries of TADs and, thereby, transitions in the spatial clustering of genome-bound CTCF. Upon entry into senescence, Zirkel et al., showed that HMGB2 is downregulated which results in profound changes in TAD boundaries and CTCF sub-nuclear localization across several cell types. This study demonstrated the important concept that CTCF can undergo changes in sub-nuclear distribution in distinct cell types under different conditions [57]. This complements our findings that a particular form of CTCF dynamically associates with SC-35-nuclear speckles in primary human epithelial cells and neuronal progenitors and is regulated by stress signals.

SC-35 nuclear speckles are proposed to have an active role in gene expression, post-transcriptional mRNA splicing, and mRNA export [50,51,92]. These nuclear bodies are further defined by their components, which include poly(A)RNAs and noncoding RNAs, as well as proteins involved in RNA synthesis and processing. Our data show that CTCF is a stress-regulated SC-35 interaction partner or nuclear speckle component in HMECs. Interestingly, CTCF has an intrinsic ability to bind large numbers of distinct RNAs through both the RNA Binding Region (RBR) and two of its 11 zinc-fingers that are not involved in DNA binding [17,20,22]. Mutations within zinc fingers 1 and 10 that abolish RNA binding also disrupt CTCF-chromatin interactions and insulator function [17]. Moreover, CTCF self-association *in vitro* is RNase-sensitive and deleting the RBR results in loss of about half of all chromatin loops in mouse embryonic stem cells by reducing CTCF-genomic binding and clustering [19]. CTCF has also been shown to sequester into nuclear “clusters” through its RNA-binding domains. This may increase the efficiency of CTCF to locate its genomic binding sites [48] and be guided by non-coding RNAs that coordinate transcription within a multi-gene chromatin domain [18]. Together, these studies support the notion that formation of a substantial number of CTCF-mediated chromatin loops requires sequestering within RNA-enriched nuclear domains to enhance RNA association through the RBR and specific zinc fingers within the DNA binding domain [17–19,48]. In addition to RNA-mediated genomic recruitment, CTCF has also been implicated in RNA processing. For example, CTCF is proposed to regulate alternative splicing by facilitating RNAP II elongation through CTCF-dependent chromatin loops that bring promoters and intragenic regions into proximity and enable exon inclusion in spliced mRNA [53]. Alternative splicing can be modulated by TET protein-dependent methylation of CTCF DNA recognition sites which affects formation of chromatin loops [52]. Interestingly, CTCF-mediated intragenic looping was shown to regulate alternative exon usage, particularly of genes involved in signaling and the stress response to presumably generate functional diversity and cellular adaptation [53].

The reported roles of CTCF in RNA processing are closely connected with its ability to regulate transcription and chromatin topology through DNA binding. This prompted us to investigate CTCF-RNA interactions to gain insight into possible CTCF activities at SC-35 nuclear speckles. Our comparative RIP-seq analyses in HMECs and vHMECs revealed hundreds of specific RNA interactions between CTCF and SC-35. Surprisingly, RNA interactions that are common to both CTCF and SC-35 in HMECs but dissociate from CTCF upon stress are implicated in cell differentiation or neuronal function. Indeed, our follow-up analysis highlights the presence and stress-sensitivity of CTCF enrichment at SC-35 nuclear speckles explicitly at the progenitor stage of neural differentiation. This is consistent with studies on early cortex formation showing a role for CTCF in regulating the balance between proliferation, differentiation and survival of neuroprogenitor cells [93]; and numerous reports demonstrating that alterations in RNA biology/processing are important mechanisms driving cellular differentiation or adaptation to stress [94–96].

Taken together, our study provides evidence that CTCF is implicated in RNA biology and tightly controlled by changes in CTCF protein stability through cellular stress signals. The observation that this occurs in specific cell types, primary HMECs and hPSC-derived neural progenitors (NPCs), highlights the physiological relevance and specialized function of CTCF at SC-35 nuclear speckles. This raises new questions about the potential role and mechanisms of action of CTCF in RNA biology, differentiation, and the stress response. Recent live cell imaging studies have revealed that gene-rich chromosomal regions dynamically position themselves near the nuclear speckle periphery where active transcription can occur [50,97]. These speckle-associated chromosome domains (SPADs) are enriched in highly expressed genes [98,99] whose regulation may be fine-tuned by the high concentration of RNA processing, modification and export factors in speckles that potentially accelerate reaction rates of generating functional RNAs [92,100]. Moreover, evidence exists for an active mechanism of localizing genes near nuclear speckles as shown by the actin-dependent movement of heat shock Hsp70HS transgenes to the nuclear speckle periphery upon heat shock induction. Importantly, the amplification of nascent transcripts from heat shock transgenes was dependent upon association with nuclear speckles [101,102].

We speculate that the function of CTCF at SC-35 nuclear speckles is to maintain cell identity in a progenitor state through alternative RNA splicing or other aspects of RNA metabolism and export. One possibility is that CTCF facilitates the active movement of select genomic domains to the nuclear speckle periphery to finely regulate expression of genes that govern cell-specific differentiation and stress-adaptability in specialized cell types. Upon stress, this function is rapidly abrogated by targeted degradation of CTCF protein that localizes to nuclear speckles, while leaving genome-occupancy by CTCF largely unaffected. The rapid destruction of particular forms of CTCF in response to stress is striking and may reveal a very critical switch in cell identity to safeguard especially vulnerable cells from stress-induced damage, potentially by releasing select genes from the nuclear speckle periphery for immediate deactivation. The wealth of studies that demonstrate CTCF is in dynamic exchange with its cofactors and the genome [44–47] support the notion that CTCF can quickly respond to environmental signals and modify its multiple functions to promote cell adaptability and survival. Future experiments should illuminate the role of CTCF in nuclear speckles by investigating: which aspects of RNA metabolism might specific forms of CTCF modulate through its interaction with SC-35 and shared RNAs; whether CTCF actively directs specific genomic regions to nuclear speckles; and why this particular CTCF activity is intimately linked to cellular stress for rapid decoupling.

Lastly, a newly appreciated area of investigation is how spatial and temporal patterns of CTCF are regulated through changes in stress-sensitive protein stability by post-translational modifications and interaction partners that modulate CTCF functions during mammary epithelial and neuronal development.

## Materials and methods

### Ethics statement

Studies using human tissue were approved by the Salk Institute Institutional Review Board (Protocol #10–0005). No human subjects were specifically recruited for our study and all patient specimens were de-identified.

### Cell culture and stress induction

Human mammary epithelial tissues were reliably procured from the UCSD Tissue Biorepository and the National Disease Research Interchange (NDRI). HMECs were propagated from reduction mammoplasty tissue from multiple, cancer-free female donors (age range 20–40 years old) chosen in an unbiased fashion. Upon notification from the surgeons and approval from the pathology test, the fresh breast tissue was either shipped on ice or picked up in person within 24 hours of surgery. Breast tissues were immediately documented for appearance, catalogued, and dissected in a laminar flow hood. In sterile 150 mm petri dishes (Thermo Fisher), crude surgery sites, fat areas and fibrotic tissue were discarded, while mammary gland-rich areas were isolated. The latter was further cut into smaller cubical-like sections, using disposable scalpels (Feather) and prepared as described [2,6,7]. In brief, the dissected material was placed into 50 ml Falcon tubes and digested overnight at 37°C in DMEM/F-12 (Invitrogen) supplemented with 10 μg/ml insulin, 10% FBS (Gibco), 200 U/ml crude collagenase (Sigma) and 100 U/ml hyaluronidase on a tube rotator (Fisher Scientific). The digested tissues were centrifuged at 600 x g for 5 minutes at room temperature. First, the upper fat-lipid section was pipetted off into another 50 ml tube for disposal, followed by the supernatant containing mostly media. The resuspended pellet containing the organoid pool was concentrated on top of a 40 μm strainer (Greiner), followed by a quality check under a light microscope. If organoids were not visibly cleaned of connective tissue, another round of digestion was performed. 100 mm tissue culture dishes were pre-rinsed with MEGM media (Lonza), followed by pipetting the organoids drop-wise in a small volume (2–5 ml) of MEGM media and in a spaced manner–leaving at least 2 cm space between organoids–to improve their attachment. Organoids were left for 10 minutes at room temperature to attach. After initial attachment, the 100 mm tissue culture dish was slowly and carefully filled up to 20 ml MEGM media without disrupting the organoid attachment to the tissue culture dish. Organoids were cultured on 100 mm tissue culture dishes in MEGM media (Lonza) for 10 days, until HMEC mitotic outgrowth was achieved. HMECs and vHMECs were maintained in MEGM media (Lonza). MCF-10A cells (ATCC) were cultured in DMEM/ F-12 supplemented with 100 ng/ml cholera toxin, 20 ng/ml epidermal growth factor (EGF), 0.01 mg/ml insulin, 500 ng/ml hydrocortisone, and 5% horse serum. All growth factors were purchased from Sigma. MDA-MB-231 and MCF-7 cells were obtained from ATCC and cultured following ATCC recommendations. hPSCs (EC#11) were derived from human umbilical vein cells (Lonza) as previously described [103] and provided by the Salk Institute Stem Cell Core for subsequent culturing and differentiation into neural progenitor cells (NPCs), and early neurons following previously established methods [73]. In brief, 6 well plates were coated with Matrigel (BD Bioscience) in DMEM-F12 overnight. hPSCs were thawed and seeded in presence of Rock inhibitor in mTeSR1 media (Stem Cell Technologies). Subsequently, media was changed daily in absence of Rock inhibitor. Colonies were monitored for areas of de-differentiation, which were marked and aspired. Once colonies were large and nearly touching in a ~70% confluent plate, colonies were detached with collagenase (Invitrogen) at 37°C until they were floating, and then collected and washed in DMEM/F12 medium. Cells were resuspended in Neural Induction Medium (NIM: containing DMEM/F12 as base, 1 x N2 Supplement (Invitrogen), 1 x B27 supplement (Invitrogen) and transferred to a 10 cm ultra-low-attachment plate. After one week of culturing, cells were transferred to a 10 cm polyornithine/laminin-coated plate in NIM supplemented with 1 μg/ml laminin. After one week, rosettes were manually isolated and plated on polyornithine/laminin-coated wells in Neural Progenitor Medium (NPM: containing DMEM/F12 as base, 1 x N2 supplements, 1 x B27 supplements, 100 ng/ml FGF8 (Peprotech), and 200 ng/ml SHH (R&D). For neural differentiation, cells were dissociated with Accutase (Innovative Cell Technologies) with vigorous pipetting and then plated on polyornithine/laminin plates at 10^4^ cells/cm^2^ in NPM. The next day, the medium was switched to Neural Differentiation Medium (NDM: containing DMEM/F12 as base, 1 x N2 Supplement (Invitrogen), 1 x B27 supplement (Invitrogen), 20 ng/ml BDNF (Peprotech), 20 ng/ml GDNF (Peprotech), 200 nM ascorbic acid (Sigma), 1 μg/ml laminin (Invitrogen). Cells were maintained in NDM for at least 4–6 weeks. Oxidative stress in cultured cells was induced as indicated by the addition H_2_O_2_ (Thermo-Fisher) to the culture media. Genotoxic stress was induced as indicated by the addition of 400 nM Doxorubicin to the culture media for 24 hours. Importantly, it is critical to use freshly prepared HMECs and low-passage aliquots of MCF10A cells to preserve “normalcy”. Each cell type rapidly undergoes a stress response accompanied by elevated ALDH1A3 and decreased CTCF levels or the appearance of multiple CTCF isoforms simply by freeze-thawing and prolonged tissue culturing.

### Antibodies

All antibodies are listed in S4 Table.

### Chromatin Immunoprecipitation (ChIP)

ChIPs were performed essentially as described [104] with minor modifications. Unless stated otherwise, all chemicals were purchased from Sigma. Briefly, normal HMECs and stressed HMECs were cultured on 150 mm tissue culture plates until cells were 60–70% confluent. After 1 x PBS wash, cells were crosslinked for 15 minutes at room temperature by addition of formaldehyde (Invitrogen) to a 1% final concentration in 1 x PBS. Crosslinking was stopped upon dropwise addition of glycine to a final concentration of 125 mM for 5 minutes. After washing cells twice with 1X PBS, cells were trypsinized and counted. 2 x 10^7^ cell/ml were collected in RIPA buffer containing protease inhibitors (150 mM NaCl, 1% NP-40, 0.5% Sodium Deoxycholate, 0.1% SDS, 50 mM Tris-HCl pH 8.0, 5 mM EDTA, 0.2 mM NaF, 0.2 mM sodium orthovanadate, 5 μM trichostatin A, and 5 mM sodium butyrate) and incubated on ice for 10 minutes. Lysates were subsequently sonicated on ice four times for 15 sec each with a small probe at medium setting to generate DNA fragments that are smaller than ~500 bp. Samples were centrifuged at 4°C, 13,000 rpm for 10 minutes. The collected supernatant was measured for DNA quality control on a nanodrop spectrophotometer (NanoDrop Technologies Inc.) and protein content was determined with a BCA kit (Pierce). 500 mg of lysate was diluted with 10 volumes of dilution buffer (1% Triton X-100, 150 mM NaCl, 2 mM EDTA pH8, 20 mM Tris pH8, 1X protease cocktail Roche) and pre-cleared by rotating for 1 hour at 4°C using 40 ml of a 50% slurry of 1:1 protein A- and protein G-Sepharose beads (GE Healthcare). Samples were centrifuged for 10 sec at 1500 rpm at 4°C to pellet the beads and remove the supernatant. For immunoprecipitation, 5 μg anti-CTCF (Millipore; 07–729) was added to precleared lysates along with 40 μl of a 50% slurry of 1:1 protein A/G beads that were preblocked with 1 mg/ml BSA and 0.3 mg/ml salmon sperm DNA followed by incubation at 4°C overnight. Immuno-complexes were washed with IP wash buffer (100 mM Tris-HCl pH 8.5, 500 mM LiCl, 1% NP-40, 1% Sodium Deoxycholate) and rotated for 5 minutes at 4°C. Samples were centrifuged as described above and supernatant was removed. Washes were repeated 2 more times with 1X TE buffer. Elution was performed in buffer containing 70 mM Tris-HCl pH 8.0, 1 mM EDTA and 1.5% SDS by incubation at 65°C for 10 or 30 minutes. Crosslinks were reversed from immuno-complexes by addition of 200 mM NaCl and incubation at 65°C for 6 hours or overnight. DNA was purified by incubation with proteinase K and phenol-chloroform extraction and a fraction was used as template in real-time PCR reactions. Primers were designed with Primer Express 2.0. PCR products range in size between 50 and 75 bp (see RT-PCR section for sequences). PCR reactions contained 1× SYBR Green Mix (Applied Biosystems), 1/100 fraction of the ChIP-enriched DNA, and 100 nM primers. Standard curves from 1–200 ng of sonicated genomic DNA was run alongside ChIP samples for each individual primer; input samples were treated identically and were used to subtract/normalize the values from ChIP samples.

### Real time PCR (RT-PCR)

For gene expression analysis, adherent cells were washed 1 X PBS, followed by addition of trypsin for 5 minutes in a 37°C cell culture incubator, with frequent checking under the microscope for cell rounding and lifting. Cells were pipetted off the plate with 10% FBS in DMEM and centrifuged to harvest the cell pellet in buffer 1 of the RNeasy kit (Qiagen) (following the manufacturer’s instructions). Next, samples were subjected to DNase (Invitrogen) treatment according to the manufacturer’s instructions. The extracted RNA concentration was measured with a nanodrop spectrophotometer (NanoDrop Technologies Inc.) and adjusted with ddH_2_O to a 0.5 mg RNA concentration. cDNA was synthesized from this 0.5 mg of RNA using Superscript III Reverse transcriptase (Invitrogen) according to the manufacturer’s protocol. In brief, PCR tubes containing 0.5 mg RNA were mixed with 1 μl Hexaprimer and 1 μl 10 mM dNTPs and were incubated at 65°C for 5 minutes. The following RT-PCR reaction was carried out as follows: 25°C for 10 minutes, 50°C for 50 minutes, 85°C for 5 minutes. cDNAs were analyzed by RT-PCR using SYBR Green and specific primers. All the selected primers had an annealing temperature of 60°C: CTCF (FWD CCCACGAGAAGCCATTCAAG, Rev GCTGGCATAACTGCACAAAC), p16 (FWD CATAGATGCCGCGGAAGGT, Rev CCCGAGGTTTCTCAGAGCCT), RASSF1A (FWD TATAGCCTGGGCAAGTCCTG, Rev GTACAGGGCGATCCACACTT), PUMA (FWD AGAGGGAGGAGTCTGGGAGTG, Rev GCAGCGCATATACAGTATCTTACAGG), p53 (FWD GAGCTGAATGAGGCCTTGGA, Rev CTGAGTCAGGCC CTTCTGTCTT), c-Myc (FWD GCCATTACCGGTTCTCCATA, Rev CAGGCGGTTCCTTAAAACAA), HDM2 (FWD GGCGATTGGAGGGTAGACCT, Rev CACATTTGCCTGGATCAGCA), and GAPDH (FWD TGCACCACCAACTGCTTAGC, Rev GGCATGGACTGTGGTCATGAG). Primer-specific PCR mix was obtained by diluting Power SYBRGreen MasterMix (Applied Biosystems) 1:1 and primers to a final concentration of 100 nM. Two μl of a 1:200 cDNA dilution was added to each well. For each experiment, samples were prepared in triplicates on MicroAmp plates (Applied Biosystems), while plates were sealed, gently mixed, and centrifuged at 2000 rpm for 1 minutes. Samples were run for 40 reaction cycles in Applied Biosystem 7900 HT Fast RT-PCR System. The curves obtained were quantified with the software SDS 2.4 (Applied Biosystems).

### Western blotting and co-immunoprecipitation

For Western blot analysis standard methodologies were used. Cells were trypsinized for 5 minutes at 37°C, washed in 1 x PBS, centrifuged at 3000 rpm for 3 min at 4°C. The pellet was collected in RIPA buffer and snap-frozen in liquid nitrogen for storage at -80°C. Samples were thawed on ice for 45–60 minutes prior to centrifugation at 10,000 rpm for 10 minutes at 4°C. The supernatant was collected, and the pellet was discarded. Protein content was determined using a BCA kit (Pierce). 20 μg protein/sample in a total volume of 25 μl RIPA buffer + 5 μl 5 x SDS loading dye were separated by electrophoresis using 4–12% Bis-Tris Plus Gels (Invitrogen). Transfer was performed on a gel transfer device (Invitrogen) with similar results using the Nitrocellulose or PVDF iBlot Transfer Stacks (Invitrogen). The following transfer program was used: 20V for 1 minutes, 23V for 3 minutes, 25V 4 minutes. Membranes were blocked in 5% skim milk in 20 mM Tris (pH 7.4)-buffered saline (150 mM NaCl) and 0.1% Tween 20 (TBST) for at least 2 hours at room temperature. Membranes were incubated using specific antibodies overnight at 4°C: rabbit anti-CTCF (Active Motif; AB_2614975; 1:1000 dilution); mouse anti-p16 (Sigma; NA29; 1:1000 dilution); rabbit anti-H2A.Z (Active Motif; 39113; 1:1000 dilution); rabbit anti-actin (Sigma; A2066; 1:5000 dilution); mouse anti-p53 (Sigma; DO-1; 1:1000 dilution); rabbit anti-ALDH1A3 (Novus; NBP2-15339; 1:2000 dilution); rabbit anti-Smad2/3 (Cell Signaling; 3102; 1:1000 dilution); rabbit anti-EZH2 (Active Motif; AB_2614956; 1:1000 dilution); rabbit anti-RNA Pol II (Millipore; clone CTD4H8; 1:1000 dilution); mouse anti-SC-35 (Novus; NB100-1774; 1:2000 dilution); mouse anti-hnRNPK (Santa Cruz: D6; 1:500 dilution); mouse anti-Nestin (Abcam; ab22035; 1:500 dilution); mouse anti-beta III Tubulin (tuj1) (Abcam; ab78078; 1:1000 dilution); rabbit anti-Nanog (Abcam; ab80892; 1:1000 dilution); and rabbit anti-Sox2 (Abcam; ab97959; 1:1000 dilution). Membranes were then washed in 1 x TBST for 1 hour at room temperature, with a buffer change every 10–15 minutes. Finally, secondary antibody in the form of either HRP-conjugated anti-rabbit (Cat. No sc-2004), or anti-mouse (Cat. No sc-2005) antibodies (1:10000 dilution) (Santa Cruz) were added to membranes for 1 hour at room temperature, followed by 1 hour washes in 1 x TBST at room temperature, changing buffer every 10–15 minutes. Membranes were incubated with ECL system (Pierce) for 30 seconds– 5 minutes at room temperature, following the manufacturers protocol and visualized with a gel documentation system (Biorad). For co-immunoprecipitation, cells were washed in 1 x PBS, trypsinized at 37°C for up to 5 minutes, collected in 10% FBS in DMEM media, counted and centrifuged at 3000 rpm for 10 min at room temperature. Cells were lysed using twice the volume of whole cell extract lysis buffer (20 mM Tris HCl pH 7.5, 420 mM NaCl, 2 mM MgCl_2_, 1 mM EDTA, 10% glycerol, 0.5% NP-40, 0.5% Triton-X-100) for 45–60 minutes on ice. Samples were centrifuged at 13,000 rpm for 10 minutes at 4°C. The supernatant was recovered, and the protein content was determined with the BCA kit. 2 mg lysate were diluted 6X with IP buffer (20 mM Tris HCl pH 7.5, 50 mM NaCl, 10 mM MgCl_2_, 2 mM EDTA, 0.5% Triton-X-100) and pre-cleared using 50 ml of protein-G Sepharose beads, rotating the samples for 2 hours at 4°C. To remove the pre-clearance beads, samples were centrifuged at 1,500 rpm for 10 seconds at 4°C and the pre-cleared lysates in the supernatant were transferred into a new tube on ice. To pre-cleared lysates, 2–5 mg of antibody was added and incubated for 1 hour at 4°C. Subsequently, a 50% slurry of protein-G beads was added and further incubated for 2–4 hours. Immunocomplexes were washed with IP buffer containing 0.5% Triton-X-100 thrice and once with IP buffer containing 0.1% Triton-X-100. In between washes, samples were rotated at 4°C, then centrifuged at 1,500 rpm for 10 seconds at 4°C. 2X SDS loading buffer was added to the centrifuged pellet containing beads and immune-precipitated samples, then boiled for 10 minutes at 95°C in a heating block before SDS-PAGE and Western blotting using the antibodies indicated above.

### Mass spectrometry

CTCF complexes were immunoprecipitated from three 80% confluent MCF-10A 15 cm dishes with either 2 mg/ml of mouse anti-CTCF (B-5; Santa Cruz) or an IgG control. The resulting samples in the form of centrifuged pellets, containing beads and immune-precipitated samples in 2X SDS loading buffer, were given to the mass spec facility where samples were prepared and analyzed according to established protocols. In brief, samples were precipitated by methanol/chloroform. Dried pellets were dissolved in 8M urea/100 mM TEAB, pH 8.5. Proteins were reduced with 5 mM Tris(2-carboxyethyl)phosphine hydrochloride (TCEP, Sigma-Aldrich) and alkylated with 10 mM chloroacetamide (Sigma-Aldrich). Proteins were digested overnight at 37°C in 2M urea/100 mM TEAB, pH 8.5, with trypsin (Promega). Digestion was quenched with formic acid, 5% final concentration. The digested samples were analyzed on a Q-Exactive mass spectrometer (Thermo). The digest was injected directly onto a 30 cm, 75 μm ID column packed with BEH 1.7 μm C18 resin (Waters). Samples were separated at a flow rate of 300 nl/minute on an nLC 1000 (Thermo). Buffers A and B were 0.1% formic acid in water and 0.1% formic acid in 90% acetonitrile, respectively. A gradient of 1–30% B over 95 minutes, an increase to 40% B over 20 minutes, an increase to 90% B over another 10 minutes and then held at 90% B for 5 minutes of washing prior to returning to 1% B was used for 140 minutes total run time. The column was re-equilibrated with 20 μl of buffer A before injection of sample. Peptides were eluted directly from the tip of the column and nanosprayed directly into the mass spectrometer by application of 2.5 kV voltage at the back of the column. The Q Exactive was operated in a data-dependent mode. Full MS scans were collected in the Orbitrap at 70K resolution with a mass range of 400 to 1800 m/z. The 10 most abundant ions per cycle were selected for MS/MS and dynamic exclusion was used with an exclusion duration of 15 seconds. Hits are listed in S1 Table, sorted by spectral count.

### Immunofluorescence

Cells were grown on coverslips (Zeiss; Cat. No 10474379) in 6 well tissue culture plates under normal culture conditions at least overnight, or until treatments were finished. At the end point, media was removed, cells were gently washed with 1 x PBS and fixed with 4% paraformaldehyde in PBS for 20 minutes at room temperature, followed by 5 washes every 10 minutes in PBS at room temperature. Cells were then permeabilized using 0.5% Triton-X-100 in PBS for 10 minutes. Cells were washed twice with 2 x PBS for 10 minutes each. Permeabilized cells were blocked with 4% BSA in PBS for 1 hour at room temperature and incubated with specific antibodies overnight at 4°C in a humidified chamber. In this chamber, 150 μl of antibody solution drops were pipetted on parafilm and inverted coverslips were placed on top, cautiously preventing air bubbles. Handling of the coverslips was best performed with standard surgical pointed forceps. Antibodies were: mouse anti-histone H3 (Millipore; clone 6.6.2; 1:400 dilution); rabbit anti-CTCF (Active Motif; AB_2614975; 1:200 dilution); mouse anti-CTCF (Santa Cruz; B-5; 1:100 dilution); mouse anti-SC-35 (Novus; NB100-1774; 1:200 dilution); rabbit anti-PAPBN1 (Invitrogen; JM11-28; 1:200 dilution); mouse anti-hnRNPK (Santa Cruz, Clone D6; 1:100 dilution); rabbit anti-NONO (generous gift from the Panda lab, Salk Institute); or mouse anti-Nucleolin (Santa Cruz; sc-13057; 1:400 dilution). Coverslips were placed with forceps in 6 well tissue culture plates and washed 5 times for 10 minutes each with PBS. Inverted cover slips were incubated with 150 μl of secondary antibody drops conjugated with Alexa 488 or Alexa 546 fluorophores (Life Technologies; A21202; A21206; 1:400 dilutions) on parafilm, in a humidified chamber and wrapped in aluminum foil for 1 hour at room temperature. Cover slips were placed back with forceps in 6 well tissue culture plates, followed by 5 washing steps in 1 x PBS for 10 minutes at room temperature, each. 150 μl of 1μg/mL of DAPI in PBS drops (Sigma) were placed on parafilm and incubated with inverted coverslips for 5 minutes at room temperature, followed by 3 quick dipping washes in ddH_2_O in a 500 ml beaker. Excess water was removed by carefully holding one edge of the cover slip on Kimwipes. Coverslips were mounted on a glass slide using Vectashield (Vector laboratories). The edges of the coverslip were sealed with clear nail polish (Sally Hansen top coat). Slides were stored in a cardboard slide tray overnight at room temperature and at 4°C for long-term storage. All images were acquired using Airyscan mode on a ZEISS 880 LSM Airyscan confocal microscope. Samples were imaged using a 63x 1.4NA objective with a pixel size of 49nm and a z-step size of 159nm. Laser power settings were 2.74 mW (561nm), 1.05 mW (488nm), and 1.11 mW (405nm), with the detector gain set to 600. For all samples, the laser power, detector gain, pixel size, and pixel dwell time (0.66 ms/pixel) were identical. All images were processed with the same “Airyscan parameter” of 7.3.

### Poly A RNA *in situ* immunofluorescence

All buffers and washes were prepared with RNase-free solutions. Cells were grown on coverslips (Zeiss; Cat. No 10474379) in 6 well tissue culture plates at least overnight, or until treatments were finished. At the end point, media was removed, cells were gently washed with 1 x PBS and fixed with 4% paraformaldehyde in PBS for 10 minutes at room temperature, followed by fixation with ice cold methanol for 10 minutes on ice, and a rehydration step in 70% EtOH for 10 minutes on ice. Cells were incubated in 1M Tris pH 8.0 in PBS for 5 minutes at room temperature, followed by two washing steps in 1 x PBS. A 150 μl solution of biotinylated oligo d(T) probes (1:1000, Promega) in hybridization buffer (5% BSA, 10% Dextran sulfate, 25% formamide de-ionized, 1 mg/ml yeast tRNA, in 2 x SSC) was placed on parafilm in a humidified chamber. Inverted coverslips were placed on the probe drops with the help of standard surgical pointed forceps. Samples were incubated for 2 hours at 37°C in a humidified chamber, followed by placing the cover slips back in a 6 well tissue culture plate and washing steps in 4 x SSC in PBS, and 2 x SSC in PBS for 10 minutes each at room temperature. Primary antibody staining with mouse anti-CTCF (Santa Cruz; B-5; 1:100 dilution) in 2 x SSC with 0.1% Triton-X-100 in PBS was carried out as inverted cover slips on 150 μl staining drops on parafilm overnight at 4°C in a humidified chamber. Cover slips were placed back in a 6 well tissue culture plate, and washed in 2 x SSC in PBS for 10 minutes at room temperature and then incubated for 1 hour at room temperature with 150 μl staining drops of Alexa Fluor 568-labeled streptavidin (Molecular Probes; S11226; 1:400 dilution) to detect the biotin-labeled poly d(T) probe; and Alexa Fluor 488 anti-mouse (Molecular Probes; A21202; 1:400 dilution) to detect CTCF, in a humidified chamber at room temperature. Cover slips were placed back in a 6 well tissue culture plate and washed five times with 2 x SSC in PBS for 10 minutes each at room temperature. 150 μl of 1 μg/ml of DAPI in PBS drops (Sigma) were placed on parafilm and incubated with inverted coverslips for 5 minutes at room temperature, followed by 3 quick dipping washes in ddH_2_O in a 500 ml beaker. Excess water was removed by carefully holding one edge of the cover slip on Kimwipes. Coverslips were mounted on a glass slide using Vectashield (Vector laboratories). The edges of the coverslip were sealed with clear nail polish (Sally Hansen top coat). Slides were stored in a cardboard slide tray overnight at room temperature and at 4°C for long-term storage. All images were acquired using Airyscan mode on a ZEISS 880 LSM Airyscan confocal microscope. Samples were imaged using a 63x 1.4NA objective with a pixel size of 49nm and a step size of 159nm. Laser power settings were 2.74 μW (561nm), 1.05 μW (488nm), and 1.11 μW (405nm), with the detector gain set to 600. For all samples, the laser power, detector gain, pixel size, and pixel dwell time (0.66 μs/pixel) were identical. All images were processed with the same “Airyscan parameter” of 7.3.

### Image quantification

Quantification was performed with Imaris software (Bitplane). Images were preprocessed with background subtraction function. “Clusters” for anti-SC-35 fluorescence signal were defined by surfaces generated with automatic threshold settings in Imaris, which makes use of K-means clustering algorithms to classify each pixel based on their intensity [105]. “Clusters” for CTCF fluorescence signal were defined similarly but with manual thresholding due to the large intensity variation. Pearson’s correlation of CTCF and SC-35 or CTCF and H3 were then measured inside and outside of the clusters. The ratio of CTCF inside:outside of clusters was similarly obtained. All raw and analyzed data together with the detailed creation parameters are hosted in Zenodo: https://doi.org/10.5281/zenodo.4081952.

### Statistical analysis

Statistical analyses were performed using Prism (GraphPad Software). The data distribution of all control and experimental groups were first examined. In analyses where all groups passed the D’agostino & Pearson normality test, we used ANOVA and Tukey’s multiple comparisons tests. Otherwise, the non-parametric Kruskal-Wallis and Dunn’s multiple comparisons tests were used. Paired t test was used for two-condition comparison (Fig 4). Normally distributed data were shown as mean ± standard error of the mean (SEM), while median [interquartile range] was reported for the rest. Outliers from intensity inside:outside of clusters were removed using robust regression and outlier removal (ROUT) method with a false discovery rate of 1% permitted [106].

### CTCF and SC-35 based RNA-immunoprecipitation sequencing (RIP-seq)

Briefly, normal HMECs and stressed HMECs were cultured in 150 mm tissue culture dishes under normal tissue culture conditions at 37°C. Cells were washed in 1 x PBS, followed by a crosslinking step for 15 minutes at room temperature by addition of 0.1% formaldehyde in 1 x PBS. Crosslinking was stopped upon dropwise addition of glycine to a final concentration of 125 mM for 5 minutes at room temperature. After washing with 1X PBS, cells were scraped and collected in RIPA (150 mM NaCl, 1% NP-40, 0.5% Sodium Deoxycholate, 0.1% SDS, 50 mM Tris-HCl pH 8.0, 5 mM EDTA) containing protease inhibitors into a 15 ml collection tube on ice. Lysates were subsequently sonicated four times for 15 sec with a small probe at medium setting on ice. Protein concentration was measured with a BCA kit (Pierce) and 500 mg of lysate was pre-cleared by rotating samples for 1 hour at 4°C using 40 μl of a 50% slurry of 1:1 protein A- and protein G-Sepharose beads (GE Healthcare). Samples were centrifuged for 15 seconds at 1500 rpm at 4°C and the supernatant with the precleared lysate was transferred into a fresh collection tube. For immunoprecipitation, 2 mg/ml anti-CTCF (Active Motif; AB_2614975), or mouse anti-SC-35 (Novus; NB100-1774) was added to precleared lysates together with 40 ml of a 50% slurry of 1:1 protein A/G beads and incubated at 4°C overnight. Samples were centrifuged for 15 seconds at 1500 rpm at 4°C and the pellet containing beads with immuno-complexes were washed with IP wash buffer (100 mM Tris-HCl pH 8.5, 500 mM LiCl, 1% NP-40, 1% Sodium Deoxycholate) for 1 h every 15 minutes at 4°C. After immunoprecipitation, CTCF- or SC-35-associated RNAs were isolated from the immunoprecipitate according to the manufacture's protocol (Wako). After elution from the beads, RNA was prepared using a miRNeasy kit (Qiagen). To remove possible genomic DNA contamination, RNase-free DNase was used during the RNA purification steps. RNA concentration was determined using a Nanodrop spectrophotometer (NanoDrop Technologies Inc.) and its integrity was ascertained by migration on a 2% agarose gel and analyzed by displaying 28S and 18S rRNA. The RNAs were analyzed using small RNA deep sequencing (summarized in S2 Table).

### Bioinformatic analysis

Libraries were sequenced by generated 100 bp paired-end reads on HiSEq. 2000 (Illumina). Sequenced reads were quality-tested using FASTQC [107] and aligned to the hg19human genome using the STAR [108] version 2.5.1b. Mapping was carried out using default parameters (up to 10 mismatches per read, and up to 9 multi-mapping locations per read). The genome index was constructed using the gene annotation supplied with the hg19 Illumina iGenomes collection and sjdbOverhang value of 100. Raw gene expression was quantified across all gene bodies, using the top-expressed isoform as proxy for gene expression, and differential gene expression was carried out using the DESeq2 [109] package version 1.14 using replicates to compute within-group dispersion. Differentially bound transcripts were defined as having a false discovery rate (FDR) <0.05 and a log2 fold change >1 when comparing two experimental conditions. Transcripts showing sc35-specific binding and CTCF-specific binding were defined as those significantly upregulated in the sc35 RIP or CTCF RIP compared to the control RNA-seq, respectively. Transcripts showing both significant binding of CTCF and SC-35 in HMECs but no significant binding in the vHMECs were annotated with HOMER [110] and tested for term overrepresentation using WebGestalt [111]. ChIP-seq analysis was carried out using HOMER findPeaks and mergePeaks subroutines using default parameters (four-fold enrichment over input control, four-fold enrichment over local tag count, Poisson p-value < 0.001, and style factor). Peaks common to replicate 1 and replicate 2 at day 1 or day 10 were kept and merged into a common peak file. Raw read counts were then assigned to peaks using HOMER annotatePeaks using all replicates from both days. Differential CTCF peaks were found using raw counts of merged peaks from both day 1 and day 10 HMECs with DESeq2 using lenient parameters of FDR < 0.05 and log2fold > 1. Normalized read densities were visualized using the UCSC genome browser [112]. Data was submitted to GEO (GSE139886). A summary of the read statistics for ChIP-seq and RIP-seq is shown in S3 Table.

## Supporting information

S1 Fig CTCF-associated nuclear clusters are absent in certain human breast cancer cell lines and patient-derived human fibroblasts. (A) Indicated cells were stained with antibodies to CTCF and SC-35 and DAPI as a DNA marker. Samples were visualized with an Airyscan microscope. (B) Indicated cell lines were treated with 100 μM H_2_O_2_ for 24 hours. Protein levels of CTCF and actin (as a control) were assessed by Western blotting.
(TIF)

S2 Fig CTCF is enriched at nuclear speckle compartments together with polyA RNA and splicing factors. (A) HMECs were stained against antibodies to CTCF and either a polyA RNA probe; antibodies to SC-35-associated nuclear speckle proteins, PAPBN1 and hnRNP-K; a paraspeckle marker, NONO; or a nucleolus marker, Nucleolin. MCF10A cells were also stained against antibodies to CTCF and SC-35. Samples were visualized using an Airyscan microscope.
(TIF)

S3 Fig Stress-induced loss of CTCF clustering is observed with CTCF antibodies recognizing either the N- or C-terminus of CTCF. (A) Diagram of N-terminal and C-terminal CTCF antibodies epitope-recognition sites and known PTM sites in CTCF. (B) Indicated cells were stained with SC-35 and CTCF (N-terminal epitope) and DAPI as a DNA marker. (C) Indicated cells were stained with H3K27Ac and CTCF (C-terminal epitope) and DAPI as a DNA marker. (D) Indicated cells were stained with a probe for PolyA RNA, CTCF (C-terminal epitope) and DAPI as a DNA marker. Samples were visualized with an Airyscan microscope.
(TIF)

S1 Table List of mass spectrometry protein interaction partners for CTCF. (PDF)

S2 Table List of RIP-seq RNA interaction partners for CTCF. Prioritization of interaction partners was listed by enrichment over control sample (log2fold).
(PDF)

S3 Table Summary of the read statistics for ChIP-seq and RIP-seq. (XLSX)

S4 Table Summary of antibodies. (PDF)
