# Supplementary figures and images for: Dynamic regulation of CTCF stability and sub-nuclear localization in response to stress
Source: PLoS Genet. 2021 Jan 7;17(1):e1009277. doi: 10.1371/journal.pgen.1009277 (PMC7790283; doi:10.1371/journal.pgen.1009277)

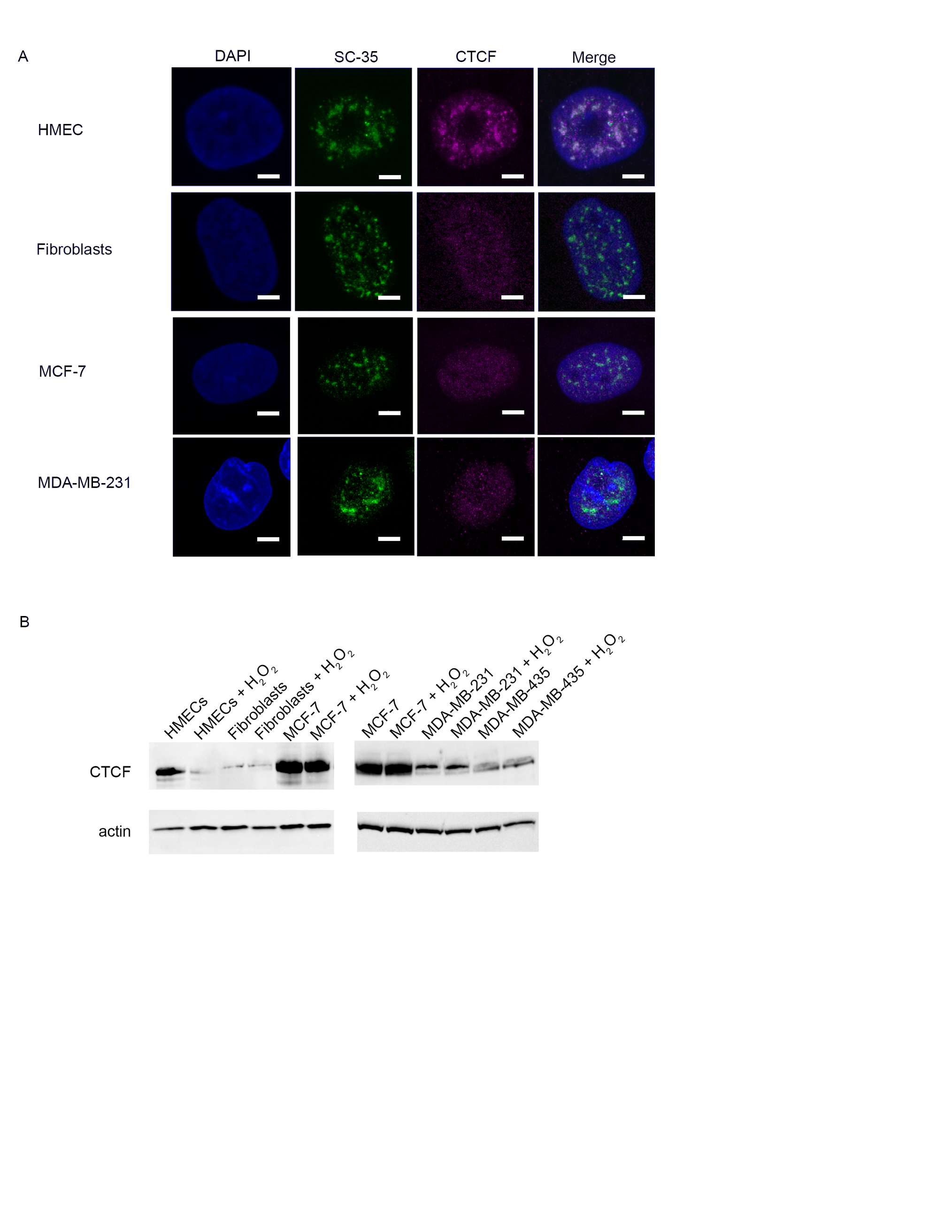

Supplement: S1 Fig — (A) Indicated cells were stained with antibodies to CTCF and SC-35 and DAPI as a DNA marker. Samples were visualized with an Airyscan microscope. (B) Indicated cell lines were treated with 100 μM H2O2 for 24 hours. Protein levels of CTCF and actin (as a control) were assessed by Western blotting. (TIF) [file pgen.1009277.s001.tif]

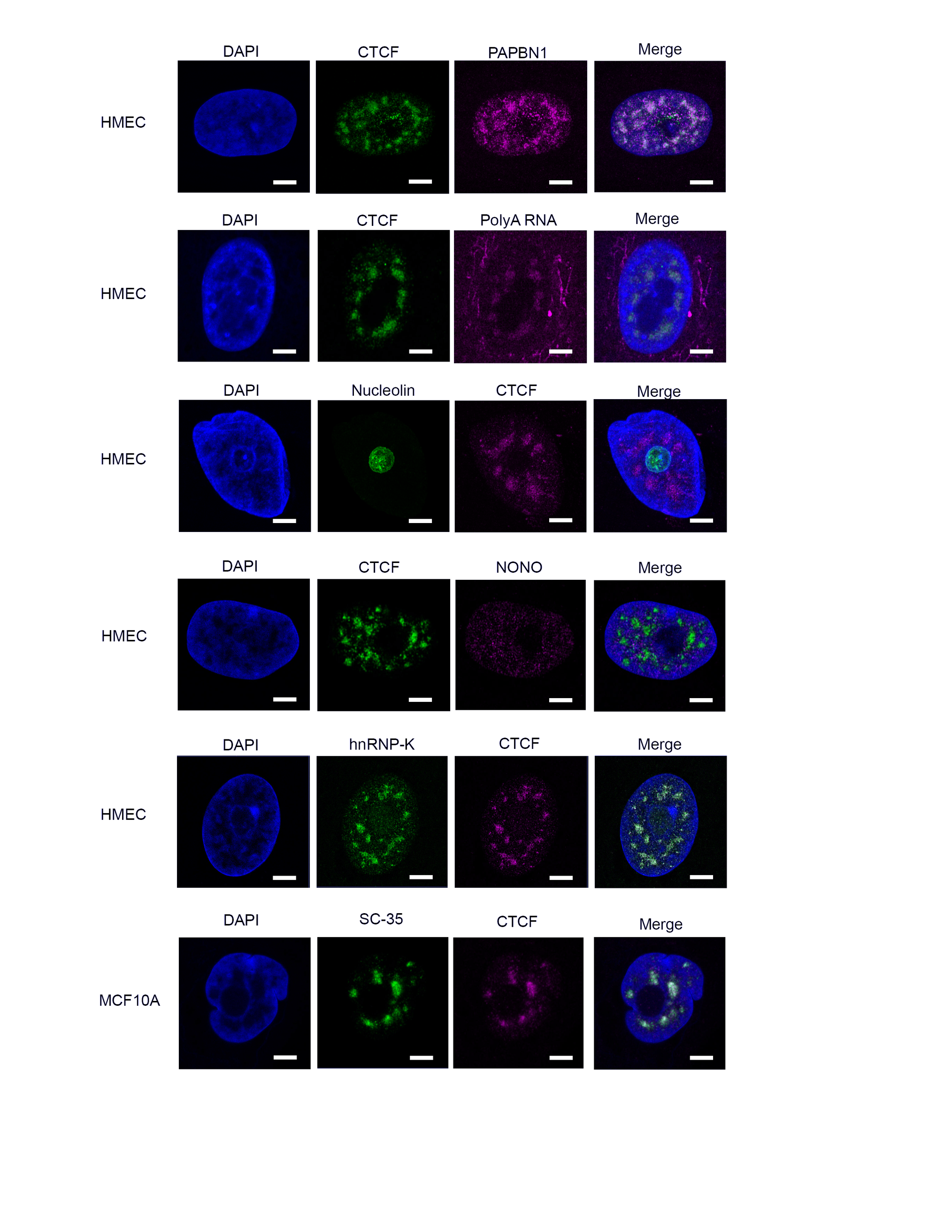

Supplement: S2 Fig — (A) HMECs were stained against antibodies to CTCF and either a polyA RNA probe; antibodies to SC-35-associated nuclear speckle proteins, PAPBN1 and hnRNP-K; a paraspeckle marker, NONO; or a nucleolus marker, Nucleolin. MCF10A cells were also stained against antibodies to CTCF and SC-35. Samples were visualized using an Airyscan microscope. (TIF) [file pgen.1009277.s002.tif]

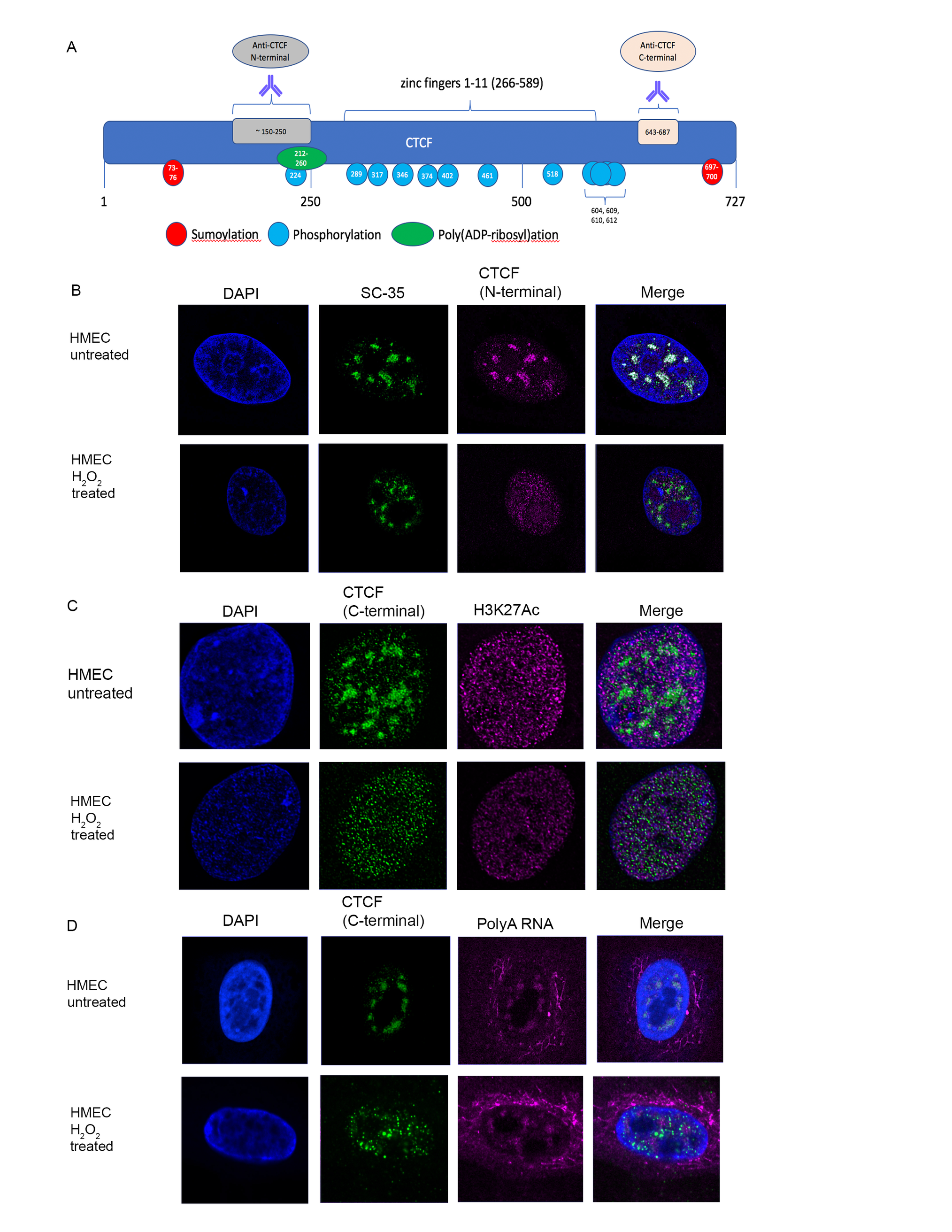

Supplement: S3 Fig — (A) Diagram of N-terminal and C-terminal CTCF antibodies epitope-recognition sites and known PTM sites in CTCF. (B) Indicated cells were stained with SC-35 and CTCF (N-terminal epitope) and DAPI as a DNA marker. (C) Indicated cells were stained with H3K27Ac and CTCF (C-terminal epitope) and DAPI as a DNA marker. (D) Indicated cells were stained with a probe for PolyA RNA, CTCF (C-terminal epitope) and DAPI as a DNA marker. Samples were visualized with an Airyscan microscope. (TIF) [file pgen.1009277.s003.tif]
