# Supplementary material for: Dynamic regulation of CTCF stability and sub-nuclear localization in response to stress
Source: PLoS Genet. 2021 Jan 7;17(1):e1009277. doi: 10.1371/journal.pgen.1009277 (PMC7790283; doi:10.1371/journal.pgen.1009277)
Supplement: S1 Table — (PDF) [file pgen.1009277.s004.pdf]

Accession	Spectral count	seqcov	Name	Description
P22626	181	74.80%	Heterogeneous nuclear ribonucleoproteins A2/B1	OS=Homo sapiens GN=HNRNPA2B1 PE=1 SV=2
P38646	106	45.80%	Stress-70 protein, mitochondrial	OS=Homo sapiens GN=HSPA9 PE=1 SV=2
P35637	86	26.00%	RNA-binding protein FUS	OS=Homo sapiens GN=FUS PE=1 SV=1
O14979	67	26.40%	Heterogeneous nuclear ribonucleoprotein D-like	OS=Homo sapiens GN=HNRNPDL PE=1 SV=3
Q00839	58	28.80%	Heterogeneous nuclear ribonucleoprotein U	OS=Homo sapiens GN=HNRNPU PE=1 SV=6
Q12906	51	23.90%	Interleukin enhancer-binding factor 3	OS=Homo sapiens GN=ILF3 PE=1 SV=3
P11021	50	34.30%	78 kDa glucose-regulated protein	OS=Homo sapiens GN=HSPA5 PE=1 SV=2
P08670	49	48.70%	Vimentin	OS=Homo sapiens GN=VIM PE=1 SV=4
P63261	46	34.70%	Actin, cytoplasmic 2	OS=Homo sapiens GN=ACTG1 PE=1 SV=1
P60709	46	34.70%	Actin, cytoplasmic 1	OS=Homo sapiens GN=ACTB PE=1 SV=1
P31943	41	37.00%	Heterogeneous nuclear ribonucleoprotein H	OS=Homo sapiens GN=HNRNPH1 PE=1 SV=4
Q14103	41	29.90%	Heterogeneous nuclear ribonucleoprotein D0	OS=Homo sapiens GN=HNRNPD PE=1 SV=1
P63267	41	26.10%	Actin, gamma-enteric smooth muscle	OS=Homo sapiens GN=ACTG2 PE=1 SV=1
P62736	41	26.00%	Actin, aortic smooth muscle	OS=Homo sapiens GN=ACTA2 PE=1 SV=1
P68133	41	26.00%	Actin, alpha skeletal muscle	OS=Homo sapiens GN=ACTA1 PE=1 SV=1
P68032	41	26.00%	Actin, alpha cardiac muscle 1	OS=Homo sapiens GN=ACTC1 PE=1 SV=1
Q99729	41	25.30%	Heterogeneous nuclear ribonucleoprotein A/B	OS=Homo sapiens GN=HNRNPAB PE=1 SV=2

P51991	41	23.80%	Heterogeneous nuclear ribonucleoprotein A3	OS=Homo sapiens GN=HNRNPA3 PE=1 SV=2
P98175	39	25.60%	RNA-binding protein 10	OS=Homo sapiens GN=RBM10 PE=1 SV=3
P05556	36	17.50%	Integrin beta-1	OS=Homo sapiens GN=ITGB1 PE=1 SV=2
Q9BUJ2	33	21.80%	Heterogeneous nuclear ribonucleoprotein U-like protein 1	OS=Homo sapiens GN=HNRNPUL1 PE=1 SV=2
P11142	31	29.10%	Heat shock cognate 71 kDa protein	OS=Homo sapiens GN=HSPA8 PE=1 SV=1
P31942	30	49.10%	Heterogeneous nuclear ribonucleoprotein H3	OS=Homo sapiens GN=HNRNPH3 PE=1 SV=2
P11940	30	17.50%	Polyadenylate-binding protein 1	OS=Homo sapiens GN=PABPC1 PE=1 SV=2
P09651	29	21.50%	Heterogeneous nuclear ribonucleoprotein A1	OS=Homo sapiens GN=HNRNPA1 PE=1 SV=5
Q12905	28	26.90%	Interleukin enhancer-binding factor 2	OS=Homo sapiens GN=ILF2 PE=1 SV=2
Q15149	28	5.10%	Plectin	OS=Homo sapiens GN=PLEC PE=1 SV=3
P26006	27	12.20%	Integrin alpha-3	OS=Homo sapiens GN=ITGA3 PE=1 SV=5
P61978	26	30.90%	Heterogeneous nuclear ribonucleoprotein K	OS=Homo sapiens GN=HNRNPK PE=1 SV=1
Q96PK6	25	27.20%	RNA-binding protein 14	OS=Homo sapiens GN=RBM14 PE=1 SV=2
P55795	23	25.80%	Heterogeneous nuclear ribonucleoprotein H2	OS=Homo sapiens GN=HNRNPH2 PE=1 SV=1
Q32P51	23	14.40%	Heterogeneous nuclear ribonucleoprotein A1-like 2	OS=Homo sapiens GN=HNRNPA1L2 PE=2 SV=2
P07910	22	26.50%	Heterogeneous nuclear ribonucleoproteins C1/C2	OS=Homo sapiens GN=HNRNPC PE=1 SV=4
P06756	21	13.00%	Integrin alpha-V	OS=Homo sapiens GN=ITGAV PE=1 SV=2

Q9H361	20	12.40%	Polyadenylate-binding protein	3 OS=Homo sapiens GN=PABPC3 PE=1 SV=2
Q92841	19	17.40%	Probable ATP-dependent RNA helicase DDX17	OS=Homo sapiens GN=DDX17 PE=1 SV=2
O00571	19	13.10%	ATP-dependent RNA helicase DDX3X	OS=Homo sapiens GN=DDX3X PE=1 SV=3
P17844	18	18.10%	Probable ATP-dependent RNA helicase DDX5	OS=Homo sapiens GN=DDX5 PE=1 SV=1
O15523	18	11.70%	ATP-dependent RNA helicase DDX3Y	OS=Homo sapiens GN=DDX3Y PE=1 SV=2
P10809	17	19.40%	60 kDa heat shock protein, mitochondrial	OS=Homo sapiens GN=HSPD1 PE=1 SV=2
O60506	17	15.20%	Heterogeneous nuclear ribonucleoprotein Q	OS=Homo sapiens GN=SYNCRIP PE=1 SV=2
Q9P2B2	17	13.70%	Prostaglandin F2 receptor negative regulator	OS=Homo sapiens GN=PTGFRN PE=1 SV=2
P52597	16	22.20%	Heterogeneous nuclear ribonucleoprotein F	OS=Homo sapiens GN=HNRNPF PE=1 SV=3
Q13310	16	13.00%	Polyadenylate-binding protein 4	OS=Homo sapiens GN=PABPC4 PE=1 SV=1
P62805	15	53.40%	Histone H4	OS=Homo sapiens GN=HIST1H4A PE=1 SV=2
P62937	15	23.60%	Peptidyl-prolyl cis-trans isomerase A	OS=Homo sapiens GN=PPIA PE=1 SV=2
O60812	15	11.60%	Heterogeneous nuclear ribonucleoprotein C-like 1	OS=Homo sapiens GN=HNRNPCL1 PE=1 SV=1
Q92804	15	11.50%	TATA-binding protein-associated factor 2N	OS=Homo sapiens GN=TAF15 PE=1 SV=1
P53992	15	8.90%	Far upstream element-binding protein 2	Protein transport protein Sec24C OS=Homo sapiens GN=SEC24C PE=1 SV=3

A5A3E0	15	3.40%	POTE ankyrin domain family member F	OS=Homo sapiens GN=POTEF PE=1 SV=2
Q6S8J3	15	3.40%	POTE ankyrin domain family member E	OS=Homo sapiens GN=POTEE PE=1 SV=3
P49711	14	10.70%	Transcriptional repressor CTCF	OS=Homo sapiens GN=CTCF PE=1 SV=1
Q4VXU2	14	6.20%	Polyadenylate-binding protein 1-like	OS=Homo sapiens GN=PABPC1L PE=2 SV=1
P04406	13	24.50%	Glyceraldehyde-3-phosphate dehydrogenase	OS=Homo sapiens GN=GAPDH PE=1 SV=3
Q9Y3I0	13	15.20%	tRNA-splicing ligase RtcB homolog	OS=Homo sapiens GN=RTCB PE=1 SV=1
Q01844	13	10.40%	RNA-binding protein EWS	OS=Homo sapiens GN=EWSR1 PE=1 SV=1
Q562R1	13	9.30%	Beta-actin-like protein 2	OS=Homo sapiens GN=ACTBL2 PE=1 SV=2
P0CG38	13	2.00%	POTE ankyrin domain family member I	OS=Homo sapiens GN=POTEI PE=3 SV=1
P52272	12	17.30%	Heterogeneous nuclear ribonucleoprotein M	OS=Homo sapiens GN=HNRNPM PE=1 SV=3
P04350	12	10.40%	Tubulin beta-4A chain	OS=Homo sapiens GN=TUBB4A PE=1 SV=2
P07437	12	10.40%	Tubulin beta chain	OS=Homo sapiens GN=TUBB PE=1 SV=2
P68371	12	10.30%	Tubulin beta-4B chain	OS=Homo sapiens GN=TUBB4B PE=1 SV=1
O43390	12	7.00%	Heterogeneous nuclear ribonucleoprotein R	OS=Homo sapiens GN=HNRNPR PE=1 SV=1
P54652	11	12.70%	Heat shock-related 70 kDa protein 2	OS=Homo sapiens GN=HSPA2 PE=1 SV=1
Q08211	11	4.60%	ATP-dependent RNA helicase A	OS=Homo sapiens GN=DHX9 PE=1 SV=4
P62979	10	39.70%	Ubiquitin-40S ribosomal protein S27a	OS=Homo sapiens GN=RPS27A PE=1 SV=2

Q5QNW6	10	31.00%	Histone H2B type 2-F	OS=Homo sapiens GN=HIST2H2BF PE=1 SV=3
Q99877	10	31.00%	Histone H2B type 1-N	OS=Homo sapiens GN=HIST1H2BN PE=1 SV=3
Q99879	10	31.00%	Histone H2B type 1-M	OS=Homo sapiens GN=HIST1H2BM PE=1 SV=3
Q99880	10	31.00%	Histone H2B type 1-L	OS=Homo sapiens GN=HIST1H2BL PE=1 SV=3
Q93079	10	31.00%	Histone H2B type 1-H	OS=Homo sapiens GN=HIST1H2BH PE=1 SV=3
P58876	10	31.00%	Histone H2B type 1-D	OS=Homo sapiens GN=HIST1H2BD PE=1 SV=2
P62807	10	31.00%	Histone H2B type 1-C/E/F/G/I	OS=Homo sapiens GN=HIST1H2BC PE=1 SV=4
P57053	10	31.00%	Histone H2B type F-S	OS=Homo sapiens GN=H2BFS PE=1 SV=2
O60814	10	31.00%	Histone H2B type 1-K	OS=Homo sapiens GN=HIST1H2BK PE=1 SV=3
Q01085	10	14.90%	Nucleolysin TIAR	OS=Homo sapiens GN=TIAL1 PE=1 SV=1
P08107	10	13.10%	Heat shock 70 kDa protein 1A/1B	OS=Homo sapiens GN=HSPA1A PE=1 SV=5
Q14764	10	11.00%	Major vault protein	OS=Homo sapiens GN=MVP PE=1 SV=4
Q9BYX7	10	7.20%	Putative beta-actin-like protein 3	OS=Homo sapiens GN=POTEKP PE=5 SV=1
P62987	9	38.30%	Ubiquitin-60S ribosomal protein L40	OS=Homo sapiens GN=UBA52 PE=1 SV=2
Q06830	9	29.60%	Peroxiredoxin-1	OS=Homo sapiens GN=PRDX1 PE=1 SV=1
P27105	9	23.30%	Erythrocyte band 7 integral membrane protein	OS=Homo sapiens GN=STOM PE=1 SV=3
P0CG47	9	21.40%	Polyubiquitin-B	OS=Homo sapiens GN=UBB PE=1 SV=1
Q92734	9	16.50%	Protein TFG	OS=Homo sapiens GN=TFG PE=1 SV=2
P06576	9	14.00%	ATP synthase subunit beta, mitochondrial	OS=Homo sapiens GN=ATP5B PE=1 SV=3

P30101	9	12.50%	Protein disulfide-isomerase A3	OS=Homo sapiens GN=PDIA3 PE=1 SV=4
P25705	9	11.00%	ATP synthase subunit alpha, mitochondrial	OS=Homo sapiens GN=ATP5A1 PE=1 SV=1
P0CG48	9	7.20%	Polyubiquitin-C	OS=Homo sapiens GN=UBC PE=1 SV=3
Q13509	9	7.10%	Tubulin beta-3 chain	OS=Homo sapiens GN=TUBB3 PE=1 SV=2
Q96KK5	8	21.90%	Histone H2A type 1-J	OS=Homo sapiens GN=HIST1H2AH PE=1 SV=3
Q9BTM1	8	21.70%	Histone H2A.J	OS=Homo sapiens GN=H2AFJ PE=1 SV=1
Q16777	8	21.70%	Histone H2A type 2-C	OS=Homo sapiens GN=HIST2H2AC PE=1 SV=4
Q8IUE6	8	21.50%	Histone H2A type 2-B	OS=Homo sapiens GN=HIST2H2AB PE=1 SV=3
Q7L7L0	8	21.50%	Histone H2A type 3	OS=Homo sapiens GN=HIST3H2A PE=1 SV=3
Q93077	8	21.50%	Histone H2A type 1-C	OS=Homo sapiens GN=HIST1H2AC PE=1 SV=3
P04908	8	21.50%	Histone H2A type 1-B/E	OS=Homo sapiens GN=HIST1H2AB PE=1 SV=2
Q6FI13	8	21.50%	Histone H2A type 2-A	OS=Homo sapiens GN=HIST2H2AA3 PE=1 SV=3
P20671	8	21.50%	Histone H2A type 1-D	OS=Homo sapiens GN=HIST1H2AD PE=1 SV=2
P0C0S8	8	21.50%	Histone H2A type 1	OS=Homo sapiens GN=HIST1H2AG PE=1 SV=2
Q96QV6	8	21.40%	Histone H2A type 1-A	OS=Homo sapiens GN=HIST1H2AA PE=1 SV=3
P16104	8	19.60%	Histone H2AX	OS=Homo sapiens GN=H2AFX PE=1 SV=2
P09012	8	18.10%	U1 small nuclear ribonucleoprotein A	OS=Homo sapiens GN=SNRPA PE=1 SV=3
P83731	8	14.60%	60S ribosomal protein L24	OS=Homo sapiens GN=RPL24 PE=1 SV=1
Q5VTE0	8	9.50%	Putative elongation factor 1-alpha-like 3	OS=Homo sapiens GN=EEF1A1P5 PE=5 SV=1
P68104	8	9.50%	Elongation factor 1-alpha 1	OS=Homo sapiens GN=EEF1A1 PE=1 SV=1

P34931	8	8.00%	Heat shock 70 kDa protein 1-like	OS=Homo sapiens GN=HSPA1L PE=1 SV=2
Q9BUF5	8	7.60%	Tubulin beta-6 chain	OS=Homo sapiens GN=TUBB6 PE=1 SV=1
P17301	8	4.60%	Integrin alpha-2	OS=Homo sapiens GN=ITGA2 PE=1 SV=1
P62241	7	19.20%	40S ribosomal protein S8	OS=Homo sapiens GN=RPS8 PE=1 SV=2
P40926	7	15.40%	Malate dehydrogenase, mitochondrial	OS=Homo sapiens GN=MDH2 PE=1 SV=3
P62917	7	14.00%	60S ribosomal protein L8	OS=Homo sapiens GN=RPL8 PE=1 SV=2
P38159	7	10.70%	RNA-binding motif protein, X chromosome	OS=Homo sapiens GN=RBMX PE=1 SV=3
Q1KMD3	7	7.90%	Heterogeneous nuclear ribonucleoprotein U-like protein 2	OS=Homo sapiens GN=HNRNPUL2 PE=1 SV=1
A6NKZ8	7	6.70%	Putative tubulin beta chain-like protein	OS=Homo sapiens PE=5 SV=2
Q3ZCM7	7	5.60%	Tubulin beta-8 chain	OS=Homo sapiens GN=TUBB8 PE=1 SV=2
A6NNZ2	7	5.60%	Tubulin beta-8 chain-like protein	OS=Homo sapiens PE=1 SV=1
Q9BVA1	7	5.60%	Tubulin beta-2B chain	OS=Homo sapiens GN=TUBB2B PE=1 SV=1
Q13885	7	5.60%	Tubulin beta-2A chain	OS=Homo sapiens GN=TUBB2A PE=1 SV=1
Q05639	7	4.30%	Elongation factor 1-alpha 2	OS=Homo sapiens GN=EEF1A2 PE=1 SV=1
Q9UPQ9	7	3.80%	Trinucleotide repeat-containing gene 6B protein	OS=Homo sapiens GN=TNRC6B PE=1 SV=4
P16403	6	23.50%	Histone H1.2	OS=Homo sapiens GN=HIST1H1C PE=1 SV=2
P10412	6	22.80%	Histone H1.4	OS=Homo sapiens GN=HIST1H1E PE=1 SV=2
P16402	6	22.60%	Histone H1.3	OS=Homo sapiens GN=HIST1H1D PE=1 SV=2

P62280	6	15.80%	40S ribosomal protein S11	OS=Homo sapiens GN=RPS11 PE=1 SV=3
P05141	6	13.80%	ADP/ATP translocase 2	OS=Homo sapiens GN=SLC25A5 PE=1 SV=7
Q15717	6	11.00%	ELAV-like protein 1	OS=Homo sapiens GN=ELAVL1 PE=1 SV=2
Q13748	6	10.90%	Tubulin alpha-3C/D chain	OS=Homo sapiens GN=TUBA3C PE=1 SV=3
Q71U36	6	10.90%	Tubulin alpha-1A chain	OS=Homo sapiens GN=TUBA1A PE=1 SV=1
P31483	6	10.90%	Nucleolysin TIA-1 isoform p40	OS=Homo sapiens GN=TIA1 PE=1 SV=3
Q9BQE3	6	10.90%	Tubulin alpha-1C chain	OS=Homo sapiens GN=TUBA1C PE=1 SV=1
P68363	6	10.90%	Tubulin alpha-1B chain	OS=Homo sapiens GN=TUBA1B PE=1 SV=1
P15880	6	7.50%	40S ribosomal protein S2	OS=Homo sapiens GN=RPS2 PE=1 SV=2
Q08380	6	6.80%	Galectin-3-binding protein	OS=Homo sapiens GN=LGALS3BP PE=1 SV=1
P07339	6	6.80%	Cathepsin D	OS=Homo sapiens GN=CTSD PE=1 SV=1
Q15436	6	6.10%	Protein transport protein Sec23A	OS=Homo sapiens GN=SEC23A PE=1 SV=2
Q9UKV8	6	4.20%	Protein argonaute-2	OS=Homo sapiens GN=AGO2 PE=1 SV=3
Q96DU9	6	2.60%	Polyadenylate-binding protein 5	OS=Homo sapiens GN=PABPC5 PE=2 SV=1
Q9H4B7	6	2.40%	Tubulin beta-1 chain	OS=Homo sapiens GN=TUBB1 PE=1 SV=1
Q9Y490	6	2.20%	Talin-1	OS=Homo sapiens GN=TLN1 PE=1 SV=3
Q09666	6	1.20%	Neuroblast differentiation-associated protein AHNAK	OS=Homo sapiens GN=AHNAK PE=1 SV=2
P07737	5	30.70%	Profilin-1	OS=Homo sapiens GN=PFN1 PE=1 SV=2
Q16778	5	21.40%	Histone H2B type 2-E	OS=Homo sapiens GN=HIST2H2BE PE=1 SV=3

P23527	5	21.40%	Histone H2B type 1-O	OS=Homo sapiens GN=HIST1H2BO PE=1 SV=3
P33778	5	21.40%	Histone H2B type 1-B	OS=Homo sapiens GN=HIST1H2BB PE=1 SV=2
P06899	5	21.40%	Histone H2B type 1-J	OS=Homo sapiens GN=HIST1H2BJ PE=1 SV=3
P06748	5	16.70%	Nucleophosmin	OS=Homo sapiens GN=NPM1 PE=1 SV=2
P62913	5	15.70%	60S ribosomal protein L11	OS=Homo sapiens GN=RPL11 PE=1 SV=2
Q15365	5	12.10%	Poly(rC)-binding protein 1	OS=Homo sapiens GN=PCBP1 PE=1 SV=2
P36578	5	10.30%	60S ribosomal protein L4	OS=Homo sapiens GN=RPL4 PE=1 SV=5
P23588	5	9.70%	Eukaryotic translation initiation factor 4B	OS=Homo sapiens GN=EIF4B PE=1 SV=2
P14618	5	8.70%	Pyruvate kinase PKM	OS=Homo sapiens GN=PKM PE=1 SV=4
F5H284	5	8.50%	Peptidyl-prolyl cis-trans isomerase A-like 4D	OS=Homo sapiens GN=PPIAL4D PE=3 SV=1
Q9Y536	5	8.50%	Peptidyl-prolyl cis-trans isomerase A-like 4A/B/C	OS=Homo sapiens GN=PPIAL4A PE=2 SV=1
Q15233	5	8.10%	Non-POU domain-containing octamer-binding protein	OS=Homo sapiens GN=NONO PE=1 SV=4
Q07666	5	7.90%	KH domain-containing, RNA-binding, signal transduction-associated protein 1	OS=Homo sapiens GN=KHDRBS1 PE=1 SV=1
Q96AE4	5	6.20%	Far upstream element-binding protein 1	OS=Homo sapiens GN=FUBP1 PE=1 SV=3
P23246	5	5.50%	Splicing factor, proline- and glutamine-rich	OS=Homo sapiens GN=SFPQ PE=1 SV=2
Q15437	5	4.20%	Protein transport protein Sec23B	OS=Homo sapiens GN=SEC23B PE=1 SV=2

Q9C0C2	5	2.40%	182 kDa tankyrase-1-binding protein	OS=Homo sapiens GN=TNKS1BP1 PE=1 SV=4
Q8NDV7	5	1.20%	Trinucleotide repeat-containing gene 6A protein	OS=Homo sapiens GN=TNRC6A PE=1 SV=2
P0CG39	5	1.00%	POTE ankyrin domain family member J	OS=Homo sapiens GN=POTEJ PE=3 SV=1
P47914	4	18.20%	60S ribosomal protein L29	OS=Homo sapiens GN=RPL29 PE=1 SV=2
P62266	4	16.10%	40S ribosomal protein S23	OS=Homo sapiens GN=RPS23 PE=1 SV=3
P39019	4	15.20%	40S ribosomal protein S19	OS=Homo sapiens GN=RPS19 PE=1 SV=2
P84243	4	14.70%	Histone H3.3	OS=Homo sapiens GN=H3F3A PE=1 SV=2
Q71DI3	4	14.70%	Histone H3.2	OS=Homo sapiens GN=HIST2H3A PE=1 SV=3
Q16695	4	14.70%	Histone H3.1t	OS=Homo sapiens GN=HIST3H3 PE=1 SV=3
P68431	4	14.70%	Histone H3.1	OS=Homo sapiens GN=HIST1H3A PE=1 SV=2
P46777	4	12.50%	60S ribosomal protein L5	OS=Homo sapiens GN=RPL5 PE=1 SV=3
Q02878	4	11.10%	60S ribosomal protein L6	OS=Homo sapiens GN=RPL6 PE=1 SV=3
P06733	4	8.80%	Alpha-enolase	OS=Homo sapiens GN=ENO1 PE=1 SV=2
P68366	4	8.70%	Tubulin alpha-4A chain	OS=Homo sapiens GN=TUBA4A PE=1 SV=1
Q9NY65	4	8.70%	Tubulin alpha-8 chain	OS=Homo sapiens GN=TUBA8 PE=1 SV=1
P07355	4	7.10%	Annexin A2	OS=Homo sapiens GN=ANXA2 PE=1 SV=2
A6NMY6	4	7.10%	Putative annexin A2-like protein	OS=Homo sapiens GN=ANXA2P2 PE=5 SV=2
P00367	4	6.30%	Glutamate dehydrogenase 1, mitochondrial	OS=Homo sapiens GN=GLUD1 PE=1 SV=2
Q6PEY2	4	6.20%	Tubulin alpha-3E chain	OS=Homo sapiens GN=TUBA3E PE=1 SV=2

P61247	4	5.30%	40S ribosomal protein S3a	OS=Homo sapiens GN=RPS3A PE=1 SV=2
P18564	4	5.10%	Integrin beta-6	OS=Homo sapiens GN=ITGB6 PE=1 SV=2
O00425	4	4.00%	Insulin-like growth factor 2 mRNA-binding protein 3	OS=Homo sapiens GN=IGF2BP3 PE=1 SV=2
P12235	4	4.00%	ADP/ATP translocase 1	OS=Homo sapiens GN=SLC25A4 PE=1 SV=4
P12236	4	4.00%	ADP/ATP translocase 3	OS=Homo sapiens GN=SLC25A6 PE=1 SV=4
P14625	4	3.70%	Endoplasmin	OS=Homo sapiens GN=HSP90B1 PE=1 SV=1
Q92542	4	3.10%	Nicastrin	OS=Homo sapiens GN=NCSTN PE=1 SV=2
P41219	4	2.60%	Peripherin	OS=Homo sapiens GN=PRPH PE=1 SV=2
Q8IXT5	4	2.30%	RNA-binding protein 12B	OS=Homo sapiens GN=RBM12B PE=1 SV=2
Q9NZB2	4	2.10%	Constitutive coactivator of PPAR-gamma-like protein 1	OS=Homo sapiens GN=FAM120A PE=1 SV=2
Q00610	4	2.10%	Clathrin heavy chain 1	OS=Homo sapiens GN=CLTC PE=1 SV=5
P08648	4	2.00%	Integrin alpha-5	OS=Homo sapiens GN=ITGA5 PE=1 SV=2
O75369	4	1.80%	Filamin-B	OS=Homo sapiens GN=FLNB PE=1 SV=2
Q92499	4	1.50%	ATP-dependent RNA helicase DDX1	OS=Homo sapiens GN=DDX1 PE=1 SV=2
Q07020	3	20.70%	60S ribosomal protein L18	OS=Homo sapiens GN=RPL18 PE=1 SV=2
P62861	3	16.90%	40S ribosomal protein S30	OS=Homo sapiens GN=FAU PE=1 SV=1
Q03135	3	15.20%	Caveolin-1	OS=Homo sapiens GN=CAV1 PE=1 SV=4
P23528	3	15.10%	Cofilin-1	OS=Homo sapiens GN=CFL1 PE=1 SV=3
P09211	3	14.80%	Glutathione S-transferase P	OS=Homo sapiens GN=GSTP1 PE=1 SV=2

Q13151	3	12.10%	Heterogeneous nuclear ribonucleoprotein A0	OS=Homo sapiens GN=HNRNPA0 PE=1 SV=1
P62906	3	12.00%	60S ribosomal protein L10a	OS=Homo sapiens GN=RPL10A PE=1 SV=2
P16401	3	11.10%	Histone H1.5	OS=Homo sapiens GN=HIST1H1B PE=1 SV=3
Q8N257	3	11.10%	Histone H2B type 3-B	OS=Homo sapiens GN=HIST3H2BB PE=1 SV=3
Q96A08	3	11.00%	Histone H2B type 1-A	OS=Homo sapiens GN=HIST1H2BA PE=1 SV=3
P40429	3	10.30%	60S ribosomal protein L13a	OS=Homo sapiens GN=RPL13A PE=1 SV=2
Q8NHW5	3	10.10%	60S acidic ribosomal protein P0-like	OS=Homo sapiens GN=RPLP0P6 PE=5 SV=1
P05388	3	10.10%	60S acidic ribosomal protein P0	OS=Homo sapiens GN=RPLP0 PE=1 SV=1
P62753	3	10.00%	40S ribosomal protein S6	OS=Homo sapiens GN=RPS6 PE=1 SV=1
Q12904	3	9.60%	Aminoacyl tRNA synthase complex-interacting multifunctional protein 1	OS=Homo sapiens GN=AIMP1 PE=1 SV=2
P14866	3	8.30%	Heterogeneous nuclear ribonucleoprotein L	OS=Homo sapiens GN=HNRNPL PE=1 SV=2
P12956	3	7.40%	X-ray repair cross-complementing protein 6	OS=Homo sapiens GN=XRCC6 PE=1 SV=2
Q96I24	3	7.30%	Far upstream element-binding protein 3	OS=Homo sapiens GN=FUBP3 PE=1 SV=2
O96019	3	7.20%	Actin-like protein 6A	OS=Homo sapiens GN=ACTL6A PE=1 SV=1
P62424	3	7.10%	60S ribosomal protein L7a	OS=Homo sapiens GN=RPL7A PE=1 SV=2
P0C0S5	3	7.00%	Histone H2A.Z	OS=Homo sapiens GN=H2AFZ PE=1 SV=2
Q71UI9	3	7.00%	Histone H2A.V	OS=Homo sapiens GN=H2AFV PE=1 SV=3

P49411	3	6.00%	Elongation factor Tu, mitochondrial	OS=Homo sapiens GN=TUFM PE=1 SV=2
P02545	3	5.90%	Prelamin-A/C	OS=Homo sapiens GN=LMNA PE=1 SV=1
Q96E39	3	4.60%	RNA binding motif protein, X-linked-like-1	OS=Homo sapiens GN=RBMXL1 PE=1 SV=1
Q92785	3	4.60%	Zinc finger protein ubi-d4	OS=Homo sapiens GN=DPF2 PE=1 SV=2
P17066	3	4.00%	Heat shock 70 kDa protein 6	OS=Homo sapiens GN=HSPA6 PE=1 SV=2
Q9BTA9	3	3.90%	WW domain-containing adapter protein with coiled-coil	OS=Homo sapiens GN=WAC PE=1 SV=3
Q8WUM4	3	3.90%	Programmed cell death 6-interacting protein	OS=Homo sapiens GN=PDCD6IP PE=1 SV=1
P49448	3	3.80%	Glutamate dehydrogenase 2, mitochondrial	OS=Homo sapiens GN=GLUD2 PE=1 SV=2
P07205	3	3.60%	Phosphoglycerate kinase 2	OS=Homo sapiens GN=PGK2 PE=1 SV=3
P00558	3	3.60%	Phosphoglycerate kinase 1	OS=Homo sapiens GN=PGK1 PE=1 SV=3
Q14697	3	3.40%	Neutral alpha-glucosidase AB	OS=Homo sapiens GN=GANAB PE=1 SV=3
O95487	3	3.20%	Protein transport protein Sec24B	OS=Homo sapiens GN=SEC24B PE=1 SV=2
P14923	3	3.20%	Junction plakoglobin	OS=Homo sapiens GN=JUP PE=1 SV=3
P17661	3	2.60%	Desmin	OS=Homo sapiens GN=DES PE=1 SV=3
Q92900	3	2.60%	Regulator of nonsense transcripts 1	OS=Homo sapiens GN=UPF1 PE=1 SV=2
Q96EY1	3	2.50%	DnaJ homolog subfamily A member 3, mitochondrial	OS=Homo sapiens GN=DNAJA3 PE=1 SV=2

P12268	3	2.50%	Inosine-5'-monophosphate dehydrogenase 2	OS=Homo sapiens GN=IMPDH2 PE=1 SV=2
Q16352	3	2.40%	Alpha-internexin	OS=Homo sapiens GN=INA PE=1 SV=2
Q9NZI8	3	2.30%	Insulin-like growth factor 2 mRNA-binding protein 1	OS=Homo sapiens GN=IGF2BP1 PE=1 SV=2
Q9Y6M1	3	2.20%	Insulin-like growth factor 2 mRNA-binding protein 2	OS=Homo sapiens GN=IGF2BP2 PE=1 SV=2
P39023	3	2.20%	60S ribosomal protein L3	OS=Homo sapiens GN=RPL3 PE=1 SV=2
P29401	3	1.90%	Transketolase	OS=Homo sapiens GN=TKT PE=1 SV=3
P07197	3	1.30%	Neurofilament medium polypeptide	OS=Homo sapiens GN=NEFM PE=1 SV=3
P46776	2	17.60%	60S ribosomal protein L27a	OS=Homo sapiens GN=RPL27A PE=1 SV=2
P23284	2	15.70%	Peptidyl-prolyl cis-trans isomerase B	OS=Homo sapiens GN=PPIB PE=1 SV=2
O43809	2	12.80%	Cleavage and polyadenylation specificity factor subunit 5	OS=Homo sapiens GN=NUDT21 PE=1 SV=1
Q02539	2	12.60%	Histone H1.1	OS=Homo sapiens GN=HIST1H1A PE=1 SV=3
P09382	2	11.10%	Galectin-1	OS=Homo sapiens GN=LGALS1 PE=1 SV=2
P62314	2	10.90%	Small nuclear ribonucleoprotein Sm D1	OS=Homo sapiens GN=SNRPD1 PE=1 SV=1
P60866	2	10.10%	40S ribosomal protein S20	OS=Homo sapiens GN=RPS20 PE=1 SV=1
P62277	2	8.60%	40S ribosomal protein S13	OS=Homo sapiens GN=RPS13 PE=1 SV=2
Q16629	2	8.40%	Serine/arginine-rich splicing factor 7	OS=Homo sapiens GN=SRSF7 PE=1 SV=1

P14174	2	7.80%	Macrophage migration inhibitory factor	OS=Homo sapiens GN=MIF PE=1 SV=4
P61313	2	7.80%	60S ribosomal protein L15	OS=Homo sapiens GN=RPL15 PE=1 SV=2
Q9UNX3	2	7.60%	60S ribosomal protein L26-like 1	OS=Homo sapiens GN=RPL26L1 PE=1 SV=1
P61254	2	7.60%	60S ribosomal protein L26	OS=Homo sapiens GN=RPL26 PE=1 SV=1
Q96EP5	2	7.10%	DAZ-associated protein 1	OS=Homo sapiens GN=DAZAP1 PE=1 SV=1
P08865	2	7.10%	40S ribosomal protein SA	OS=Homo sapiens GN=RPSA PE=1 SV=4
P46778	2	6.90%	60S ribosomal protein L21	OS=Homo sapiens GN=RPL21 PE=1 SV=2
Q6NXT2	2	6.70%	Histone H3.3C	OS=Homo sapiens GN=H3F3C PE=1 SV=3
P56539	2	6.60%	Caveolin-3	OS=Homo sapiens GN=CAV3 PE=1 SV=1
P46782	2	6.40%	40S ribosomal protein S5	OS=Homo sapiens GN=RPS5 PE=1 SV=4
P46781	2	6.20%	40S ribosomal protein S9	OS=Homo sapiens GN=RPS9 PE=1 SV=3
P26599	2	5.80%	Polypyrimidine tract-binding protein 1	OS=Homo sapiens GN=PTBP1 PE=1 SV=1
P22061	2	5.70%	Protein-L-isoaspartate(D-aspartate) O-methyltransferase	OS=Homo sapiens GN=PCMT1 PE=1 SV=4
Q02543	2	5.70%	60S ribosomal protein L18a	OS=Homo sapiens GN=RPL18A PE=1 SV=2
P50914	2	5.60%	60S ribosomal protein L14	OS=Homo sapiens GN=RPL14 PE=1 SV=4
P32119	2	5.60%	Peroxiredoxin-2	OS=Homo sapiens GN=PRDX2 PE=1 SV=5
P84103	2	5.50%	Serine/arginine-rich splicing factor 3	OS=Homo sapiens GN=SRSF3 PE=1 SV=1

Q5JQF8	2	5.50%	Polyadenylate-binding protein 1-like 2	OS=Homo sapiens GN=PABPC1L2A PE=2 SV=1
P36957	2	5.50%	Dihydrolipoyllysine-residue succinyltransferase component of 2-oxoglutarate dehydrogenase complex, mitochondrial	OS=Homo sapiens GN=DLST PE=1 SV=4
P04792	2	4.90%	Heat shock protein beta-1	OS=Homo sapiens GN=HSPB1 PE=1 SV=2
P27635	2	4.70%	60S ribosomal protein L10	OS=Homo sapiens GN=RPL10 PE=1 SV=4
P17987	2	4.50%	T-complex protein 1 subunit alpha	OS=Homo sapiens GN=TCP1 PE=1 SV=1
P21926	2	4.40%	CD9 antigen	OS=Homo sapiens GN=CD9 PE=1 SV=4
P26038	2	4.20%	Moesin	OS=Homo sapiens GN=MSN PE=1 SV=3
Q96PU8	2	4.10%	Protein quaking	OS=Homo sapiens GN=QKI PE=1 SV=1
O43823	2	3.80%	A-kinase anchor protein 8	OS=Homo sapiens GN=AKAP8 PE=1 SV=1
Q15366	2	3.80%	Poly(rC)-binding protein 2	OS=Homo sapiens GN=PCBP2 PE=1 SV=1
P57721	2	3.80%	Poly(rC)-binding protein 3	OS=Homo sapiens GN=PCBP3 PE=2 SV=2
P08579	2	3.60%	U2 small nuclear ribonucleoprotein B	OS=Homo sapiens GN=SNRPB2 PE=1 SV=1
P48509	2	3.60%	CD151 antigen	OS=Homo sapiens GN=CD151 PE=1 SV=3
Q86X55	2	3.60%	Histone-arginine methyltransferase CARM1	OS=Homo sapiens GN=CARM1 PE=1 SV=3
O43707	2	3.50%	Alpha-actinin-4	OS=Homo sapiens GN=ACTN4 PE=1 SV=2

Q9BS26	2	3.40%	Endoplasmic reticulum resident protein 44	OS=Homo sapiens GN=ERP44 PE=1 SV=1
Q9ULX6	2	3.40%	A-kinase anchor protein 8-like	OS=Homo sapiens GN=AKAP8L PE=1 SV=3
P19338	2	3.40%	Nucleolin	OS=Homo sapiens GN=NCL PE=1 SV=3
P14678	2	3.30%	Small nuclear ribonucleoprotein-associated proteinsB	OS=Homo sapiens GN=SNRPB PE=1 SV=2
P63162	2	3.30%	Small nuclear ribonucleoprotein-associated protein N	OS=Homo sapiens GN=SNRPN PE=1 SV=1
Q00325	2	3.30%	Phosphate carrier protein, mitochondrial	OS=Homo sapiens GN=SLC25A3 PE=1 SV=2
Q01650	2	3.20%	Large neutral amino acids transporter small subunit 1	OS=Homo sapiens GN=SLC7A5 PE=1 SV=2
O43148	2	2.90%	mRNA cap guanine-N7 methyltransferase	OS=Homo sapiens GN=RNMT PE=1 SV=1
Q5VWX1	2	2.90%	KH domain-containing, RNA-binding, signal transduction-associated protein 2	OS=Homo sapiens GN=KHDRBS2 PE=1 SV=1
Q15532	2	2.90%	Protein SSXT	OS=Homo sapiens GN=SS18 PE=1 SV=3
P50454	2	2.90%	Serpin H1	OS=Homo sapiens GN=SERPINH1 PE=1 SV=2
P52756	2	2.60%	RNA-binding protein 5	OS=Homo sapiens GN=RBM5 PE=1 SV=2
Q13148	2	2.40%	TAR DNA-binding protein 43	OS=Homo sapiens GN=TARDBP PE=1 SV=1
P13489	2	2.40%	Ribonuclease inhibitor	OS=Homo sapiens GN=RNH1 PE=1 SV=2
P08195	2	2.40%	4F2 cell-surface antigen heavy chain	OS=Homo sapiens GN=SLC3A2 PE=1 SV=3

Q12849	2	2.30%	G-rich sequence factor 1	OS=Homo sapiens GN=GRSF1 PE=1 SV=3
Q13838	2	2.30%	Spliceosome RNA helicase DDX39B	OS=Homo sapiens GN=DDX39B PE=1 SV=1
O00148	2	2.30%	ATP-dependent RNA helicase DDX39A	OS=Homo sapiens GN=DDX39A PE=1 SV=2
Q15084	2	2.30%	Protein disulfide-isomerase A6	OS=Homo sapiens GN=PDIA6 PE=1 SV=1
P26641	2	2.30%	Elongation factor 1-gamma	OS=Homo sapiens GN=EEF1G PE=1 SV=3
Q5JTV8	2	2.10%	Torsin-1A-interacting protein 1	OS=Homo sapiens GN=TOR1AIP1 PE=1 SV=2
P49368	2	2.00%	T-complex protein 1 subunit gamma	OS=Homo sapiens GN=CCT3 PE=1 SV=4
O60524	2	1.90%	Nuclear export mediator factor NEMF	OS=Homo sapiens GN=NEMF PE=1 SV=4
O15027	2	1.90%	Protein transport protein Sec16A	OS=Homo sapiens GN=SEC16A PE=1 SV=3
P02786	2	1.80%	Transferrin receptor protein 1	OS=Homo sapiens GN=TFRC PE=1 SV=2
P78362	2	1.70%	SRSF protein kinase 2	OS=Homo sapiens GN=SRPK2 PE=1 SV=3
P78332	2	1.50%	RNA-binding protein 6	OS=Homo sapiens GN=RBM6 PE=1 SV=5
P35222	2	1.30%	Catenin beta-1	OS=Homo sapiens GN=CTNNB1 PE=1 SV=1
Q9H0D6	2	1.30%	5'-3' exoribonuclease 2	OS=Homo sapiens GN=XRN2 PE=1 SV=1
Q14157	2	1.30%	Ubiquitin-associated protein 2-like	OS=Homo sapiens GN=UBAP2L PE=1 SV=2
Q5VTR2	2	1.10%	E3 ubiquitin-protein ligase BRE1A	OS=Homo sapiens GN=RNF20 PE=1 SV=2
P41252	2	0.80%	Isoleucine--tRNA ligase, cytoplasmic	OS=Homo sapiens GN=IARS PE=1 SV=2
Q14C86	2	0.70%	GTPase-activating protein and VPS9 domain-containing protein 1	OS=Homo sapiens GN=GAPVD1 PE=1 SV=2

P15924	2	0.40%	Desmoplakin	OS=Homo sapiens GN=DSP PE=1 SV=3
