# Supplementary material for: Dynamic regulation of CTCF stability and sub-nuclear localization in response to stress
Source: PLoS Genet. 2021 Jan 7;17(1):e1009277. doi: 10.1371/journal.pgen.1009277 (PMC7790283; doi:10.1371/journal.pgen.1009277)
Supplement: S2 Table — Prioritization of interaction partners was listed by enrichment over control sample (log2fold). (PDF) [file pgen.1009277.s005.pdf]

Table S2 RIPseq list of CTCF interaction partners. Prioritization of interaction partners was listed by enrichment over control sample (log2fold).

NM_ Refseq record	Gene name
NM_000094	COL7A1
NM_000203	IDUA
NM_000635	RFX2
NM_000661	RPL9
NM_000718	CACNA1B
NM_000723	CACNB1
NM_000982	RPL21
NM_001002836	ZNF787
NM_001005336	DNM1
NM_001005373	LRSAM1
NM_001008401	ZNF761
NM_001012455	ZSCAN23
NM_001013836	MAD1L1
NM_001014	RPS10
NM_001014283	DCUN1D2
NM_001017529	PRR5
NM_001024593	MSS51
NM_001025239	TSPAN4
NM_001025930	TTLL3
NM_001040057	FAM133B
NM_001080419	UNK
NM_001083111	GNRH1
NM_001083914	CTBP2
NM_001097615	POLR2J3
NM_001098537	PNPLA7
NM_001104548	MIR205HG
NM_001104595	FAM118A
NM_001126123	ENOSF1
NM_001130111	ABHD17A
NM_001134398	VAV2
NM_001135023	ELMOD3
NM_001135772	LRP5L

NM_001136537	BTBD19
NM_001136538	ACAD10
NM_001142287	SEMA4D
NM_001142397	CTIF
NM_001142405	PRELID3A
NM_001142641	FBRSL1
NM_001143962	CAPN8
NM_001144063	OSBPL5
NM_001145664	RFX8
NM_001145784	BORCS8
NM_001163692	UBAP1L
NM_001164315	ANKRD36
NM_001164759	PRKAR1B
NM_001166136	EVC2
NM_001166167	NEK6
NM_001167880	LHPP
NM_001172663	RAB40C
NM_001173539	BANP
NM_001177701	IFT27
NM_001185080	CLDN15
NM_001190328	YJEFN3
NM_001190413	RPH3AL
NM_001190707	ALS2CL
NM_001190946	FAM193B
NM_001195082	TEX22
NM_001198962	ECHDC2
NM_001199021	SPON2
NM_001201472	CORO7
NM_001201479	CORO7-PAM16
NM_001202550	WDR27
NM_001204364	FAM24B
NM_001204478	TVP23C-CDRT4
NM_001242713	LOC100289561
NM_001242786	BRF1
NM_001243279	ACSF3
NM_001243540	CEP295NL

NM_001244705	CSAD
NM_001251888	ASPSCR1
NM_001253792	ZNF444
NM_001256106	CD101
NM_001271223	OBSCN
NM_001271893	TWIST2
NM_001271943	TFEB
NM_001276274	TMEM80
NM_001277285	IGSF9B
NM_001282112	TOP3B
NM_001284303	TBC1D22A
NM_001286167	FANCA
NM_001287249	D2HGDH
NM_001291478	AKAP8L
NM_001297419	RAD52
NM_001301778	SS18L1
NM_001303012	PLCH2
NM_001303100	ERICH1
NM_001304332	PPFIA4
NM_001304808	BRD1
NM_001307939	ARID3B
NM_001308208	ARHGAP39
NM_001308281	UNC45B
NM_001308375	ANKMY1
NM_001316984	FAM207A
NM_001316994	SPACA6
NM_001317136	PFKFB4
NM_001317934	UVSSA
NM_001318766	ATG16L2
NM_001318852	MAPK8IP3
NM_001318878	STK32C
NM_001319243	RILPL1
NM_001320595	DDX5
NM_001320821	RASA3
NM_001326417	NM_001326417
NM_001409	MEGF6

NM_001670	ARVCF
NM_001794	CDH4
NM_001835	CLTCL1
NM_001932	MPP3
NM_002149	HPCAL1
NM_002386	MC1R
NM_002405	MFNG
NM_002744	PRKCZ
NM_002802	PSMC1
NM_003057	SLC22A1
NM_003086	SNAPC4
NM_003119	SPG7
NM_003185	TAF4
NM_003395	WNT9A
NM_003954	MAP3K14
NM_004076	CRYBB3
NM_004256	SLC22A13
NM_004259	RECQL5
NM_004592	SFSWAP
NM_004603	STX1A
NM_005224	ARID3A
NM_005270	GLI2
NM_005632	CAPN15
NM_005688	ABCC5
NM_005777	RBM6
NM_005781	TNK2
NM_005876	SPEG
NM_005993	TBCD
NM_006031	PCNT
NM_006037	HDAC4
NM_006092	NOD1
NM_006179	NTF4
NM_006340	BAIAP2
NM_006532	ELL
NM_006631	ZNF266
NM_006772	SYNGAP1

NM_007056	CLASRP
NM_007215	POLG2
NM_013397	PRICKLE4
NM_013398	ZNF224
NM_014067	MACROD1
NM_014068	PSORS1C1
NM_014351	SULT4A1
NM_014649	SAFB2
NM_014650	ZNF432
NM_014675	CROCC
NM_014700	RAB11FIP3
NM_014718	CLSTN3
NM_014775	SFI1
NM_014850	SRGAP3
NM_015015	KDM4B
NM_015102	NPHP4
NM_015117	ZC3H3
NM_015241	MICAL3
NM_015321	CRTC1
NM_015404	DFNB31
NM_015490	SEC31B
NM_015666	MTG2
NM_015711	GLTSCR1
NM_015981	CAMK2A
NM_016030	TRAPPC12
NM_016069	PAM16
NM_016155	MMP17
NM_016328	GTF2IRD1
NM_016642	SPTBN5
NM_017438	SETD4
NM_017566	KLHDC4
NM_017820	EXD3
NM_017891	C1orf159
NM_018032	LUC7L
NM_018140	CEP72
NM_018163	DNAJC17

NM_018688	BIN3
NM_019121	PPP1R37
NM_020133	AGPAT4
NM_020223	FAM20C
NM_020461	TUBGCP6
NM_020777	SORCS2
NM_020798	USP35
NM_021138	TRAF2
NM_021904	GABBR1
NM_022779	DDX31
NM_023011	UPF3A
NM_024653	PRKRIP1
NM_024757	EHMT1
NM_024786	ZDHHC11
NM_024821	CCDC134
NM_025176	NINL
NM_025190	ANKRD36B
NM_025225	PNPLA3
NM_032107	L3MBTL1
NM_032134	QRICH2
NM_032432	ABLIM2
NM_032450	MROH1
NM_032601	MCEE
NM_032779	CCDC142
NM_032788	ZNF514
NM_032888	COL27A1
NM_033199	UCN2
NM_033215	PPP1R3F
NM_033547	INTS4
NM_052920	KLHL29
NM_052925	LENG8
NM_052957	ACRC
NM_080722	ADAMTS14
NM_130464	NPIPB3
NM_133478	SLC4A5
NM_138399	TMEM44

NM_138422	ADAT3
NM_138465	GLI4
NM_138496	CYHR1
NM_138783	ZNF653
NM_139075	TPCN2
NM_144566	ZNF700
NM_144666	DNHD1
NM_144716	CCDC12
NM_145003	TSNARE1
NM_145057	CDC42EP5
NM_145249	IFI27L1
NM_145301	TVP23C
NM_152222	RELT
NM_152287	ZNF276
NM_152328	ADSSL1
NM_152333	SLC25A29
NM_152544	TRMT44
NM_153451	ORAOV1
NM_153813	ZFPM1
NM_173044	IL18BP
NM_173193	KCNIP2
NM_175629	DNMT3A
NM_176891	IFNE
NM_178238	PILRB
NM_178344	C3orf35
NM_178831	GATS
NM_178863	KCTD13
NM_181334	PRR5-ARHGAP8
NM_181335	ARHGAP8
NM_182924	MICALL2
NM_183378	OVCH1
NM_194448	CLEC4A
NM_198082	CCDC57
NM_198229	RGS12
NM_198406	PAQR6
NM_206861	TACC2

NM_206967	C16orf74
NM_207311	CCDC64
NM_207396	RNF207
NM_213603	ZNF789
NR_002184	RRP7BP
NR_002217	PMS2CL
NR_002594	SLC7A5P2
NR_002728	KCNQ1OT1
NR_002785	GNAS-AS1
NR_002817	AQP7P1
NR_002934	SCART1
NR_003017	SNORA71C
NR_003111	RPL32P3
NR_003148	TPM3P9
NR_003228	AFG3L1P
NR_003264	SDHAP1
NR_003265	SDHAP2
NR_003290	EP400NL
NR_003505	PPP4R1L
NR_003530	MEG3
NR_003610	PDXDC2P
NR_003659	WASH3P
NR_003950	ZDHHC8P1
NR_023383	DTX2P1-UPK3BP1-PMS2P11
NR_023386	CROCCP3
NR_024149	MEG8
NR_024237	LOC100132111
NR_024252	FAM86HP
NR_024341	EHMT1-IT1
NR_024386	PLEKHM1P1
NR_024448	GUSBP11
NR_024458	TPT1-AS1
NR_024569	LOC100130872
NR_024586	KMT2E-AS1
NR_026675	CRYM-AS1
NR_026752	CROCCP2

NR_026863	LINC00313
NR_026899	LOC146880
NR_026911	RPL21P28
NR_026922	LOC150776
NR_026947	C1RL-AS1
NR_026950	LOC283922
NR_027033	MIRLET7BHG
NR_027154	SMG1P1
NR_027155	SMG1P3
NR_027241	SNHG17
NR_027307	MEF2BNB-MEF2B
NR_027334	MZF1-AS1
NR_027409	GOLGA8A
NR_027410	GOLGA8B
NR_027460	RRN3P3
NR_027487	LOC146880
NR_027788	ZNF767P
NR_028059	PMS2P3
NR_028444	PDIA5
NR_029376	LOC100294362
NR_030343	MIR612
NR_031756	MIR711
NR_033308	RAB17
NR_033320	MIAT
NR_033396	SLC22A20
NR_033645	LMF1
NR_033825	KLHL22
NR_033854	LOC283335
NR_033967	SLC2A1-AS1
NR_034082	LOC100130950
NR_034169	FAM133DP
NR_036512	LOC100129917
NR_036569	STAG3L5P-PVRIG2P-PILRB
NR_036573	LOC155060
NR_036632	GOLGA2P5
NR_037642	LHX4-AS1

NR_037868	SLFNL1-AS1
NR_037912	CUZD1
NR_037915	FAM24B-CUZD1
NR_038198	PBX4
NR_038286	PSMD6-AS2
NR_038366	HOTAIRM1
NR_038378	LOC441242
NR_038396	TMEM147-AS1
NR_038915	PRR7-AS1
NR_038967	LOC100630923
NR_039891	MIR3064
NR_039920	MIR4763
NR_039969	MIR5047
NR_040252	ANKS3
NR_045011	SNX29P1
NR_045675	ARHGAP22
NR_047665	EPS15L1
NR_049730	ASB16-AS1
NR_049852	MIR5587
NR_102281	KCTD21-AS1
NR_103509	PHYKPL
NR_103720	STAG3L5P
NR_103728	PVRIG2P
NR_103783	BLACAT1
NR_103844	NPTN-IT1
NR_104029	DGUOK-AS1
NR_104051	CCDC84
NR_104133	ADAMTSL4-AS1
NR_104192	MIR381HG
NR_104241	TRMU
NR_104583	CALML4
NR_105059	CARMN
NR_109829	CCBL1
NR_109909	CTC-338M12.4
NR_110037	LOC100996437
NR_110056	ERI3-IT1

NR_110220	LINC01237
NR_110326	MAP3K14-AS1
NR_110473	LINC-PINT
NR_110536	CHKB-AS1
NR_110616	LINC01355
NR_110997	LOC553103
NR_111973	GS1-124K5.11
NR_111989	PGS1
NR_122105	LINC00969
NR_125365	LOC401585
NR_125375	LOC100288152
NR_126343	LOC171391
NR_126418	LENG8-AS1
NR_130745	LOC100288778
NR_131012	NEAT1
NR_132742	MIR34AHG
NR_132983	MIR193BHG
NR_132988	LOC106660606
NR_134983	PABPC1L
NR_135247	MRPS25
NR_135299	C1orf132
NR_136323	NR_136323
NR_136514	NR_136514
NR_136540	NR_136540
NR_136593	NR_136593
NR_138145	NR_138145
NR_144355	NR_144355
NR_144394	NR_144394
