# Supplementary material for: Dynamic regulation of CTCF stability and sub-nuclear localization in response to stress
Source: PLoS Genet. 2021 Jan 7;17(1):e1009277. doi: 10.1371/journal.pgen.1009277 (PMC7790283; doi:10.1371/journal.pgen.1009277)
Supplement: S4 Table — (PDF) [file pgen.1009277.s007.pdf]

Table S4. List of antibodies

Antibody	Company	Species	Mono/Poly	Clone number/ref
**CTCF**	Millipore	rabbit	Polyclonal	07-729; (raised against peptide corresponding to amino acids 659-675 near the C-terminus of human CTCF)
**CTCF**	Active Motif	rabbit	Polyclonal	RRID; AB_2614975: (raised against peptide within the N-terminal region of human CTCF. Quote Active Motif technical support: “Unfortunately I cannot provide you with the exact sequence of the immunogen, as we consider that information to be proprietary. I can disclose that the immunogen falls between peptides 150 and 250 of human CTCF.”)
**CTCF**	Santa Cruz	mouse	Monoclonal	B-5; (raised against epitope in the region 643-687 near the C-terminus of human CTCF)
**CTCF (only used in rebuttal)**	Thermo Fisher (Pierce)	rabbit	Polyclonal	Synthetic peptide corresponding to the carboxy terminus of the human CTCF protein (personal conversation technical support: “around position 657”)
**p16**	Sigma	mouse	Monoclonal	NA29
**H2A.Z**	Active Motif	rabbit	Polyclonal	Cat. No 39113
**actin**	Sigma	rabbit	Polyclonal	Cat. No A2066
**p53**	Sigma	mouse	Monoclonal	Clone DO-1
**ALDH1A3**	Novus	rabbit	Polyclonal	Cat. No NBP2-15339
**Smad 2/3**	Cell Signaling	rabbit	Polyclonal	Cat. No 3102

**EZH.2**	Active Motif	rabbit	Polyclonal	AB_2614956
**RNA Pol II**	Millipore	rabbit	Polyclonal	clone CTD4H8
**SC-35**	Novus	mouse	Monoclonal	Cat. No NB100-1774
**hnRNPK**	Santa Cruz	mouse	Monoclonal	Clone D6
**Nestin**	Abcam	mouse	Monoclonal	Cat. No ab22035
**tuj1**	Abcam	mouse	Monoclonal	Cat. No ab78078
**Nanog**	Abcam	rabbit	Polyclonal	Cat. No ab80892
**Sox2**	Abcam	rabbit	Polyclonal	Cat. No ab97959
**Histone H3**	Millipore	mouse	Monoclonal	clone 6.6.2
**PAPBN1**	Invitrogen	rabbit	Monoclonal	clone JM11-28
**Nucleolin**	Santa Cruz	mouse	Monoclonal	sc-13057
**Anti-mouse-HRP conj.**	Santa Cruz	goat	Polyclonal	Cat. No sc-2005
**Anti-rabbit-HRP conj.**	Santa Cruz	goat	Polyclonal	Cat. No sc-2004
**Anti-rabbit Alexa 546**	Life Technologies	donkey	Polyclonal	Cat. No A21206

**Anti-mouse Alexa 488**	Life Technologies	donkey	Polyclonal	Cat. No A21202
